# Hybrid EfficientNet B4 and SVM framework for rapid and accurate bone cancer diagnosis from X-rays

**DOI:** 10.1038/s41598-026-38801-3

**Published:** 2026-03-03

**Authors:** Nashaat M. Hussain Hassan, Ahmed S. Bayoumy, Mohamed Hassan M. Mahmoud

**Affiliations:** 1 Electronics and Communication Engineering Dept, Fayoum University, Fayoum, 63514 Egypt; 2 Physics and Engineering Mathematics Department, Faculty of Engineering, Kafrelsheikh University, Kafrelsheikh, 33516 Egypt; 3 Giza Institute for Higher Education and Technology, Giza, Egypt

**Keywords:** Bone cancer, Biomedical engineering

## Abstract

The early and correct diagnosis of bone cancer is important for treating both primary and metastatic conditions effectively. Traditional imaging techniques, like CT, MRI, and X-ray scans, depend exclusively on manual review, which is time-consuming and prone to human errors. Recently, ML and DL have enabled automated diagnostic systems that are more accurate, reliable, and efficient. Still, many of the existing approaches using DL suffer from high computational complexity, overfitting, and limited availability of robust datasets. This work proposes a novel diagnostic model for bone cancer, called OsteoCancerNet, which combines EfficientNetB4 for feature extraction with a support vector machine using the RBF kernel for classification. EfficientNetB4 captures efficiently both quantitative and qualitative features from X-ray images, and the SVM ensures robust binary classification. Extensive experiments using a large dataset with 29,952 X-ray images demonstrate that OsteoCancerNet provides 98% precision, 97.47% recall, 98% accuracy, and a 98% F1-score, thus outperforming traditional machine learning, deep learning, and transfer learning methods. Of note, the model maintains fast inference times of 41 milliseconds per image, making it suitable for real-time clinical applications. By combining deep learning feature extraction with traditional machine learning classification, OsteoCancerNet provides an efficient, accurate, and practical approach for the early detection of bone cancer. This approach has the potential to aid radiologists in timely diagnosis, decrease workload, and improve treatment outcomes, thus underlining the advantages of integrating DL and ML techniques within medical imaging. Keywords: OsteoCancerNet; computer-assisted diagnosis; bone cancer diagnosis; EfficientNet B4 model; SVM model; X-ray image analysis.

## Introduction

**Fig. 1:**
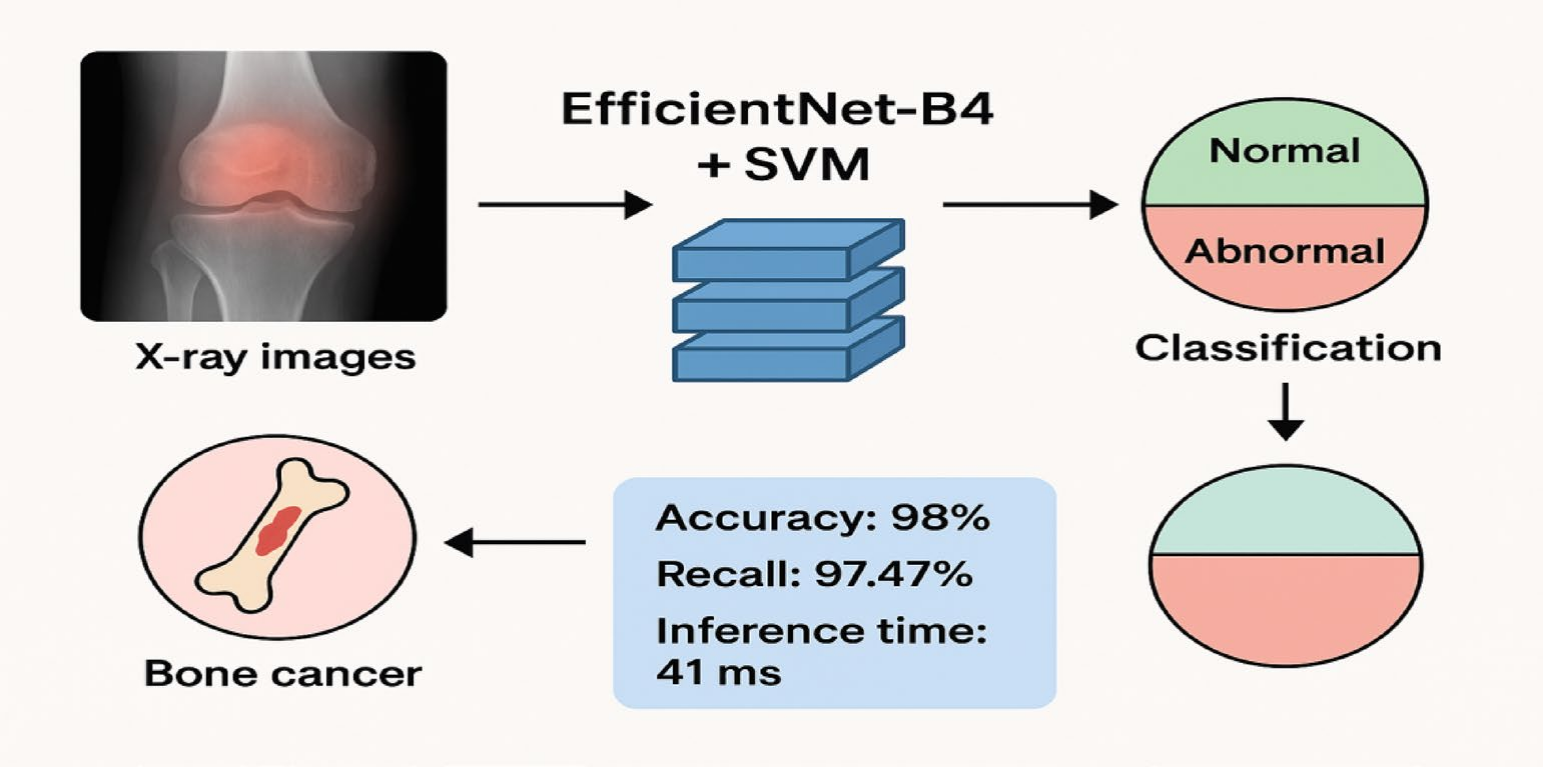
Proposed hybrid efficientnet-B4 + SVM framework for automated bone cancer detection.

 Bone cancer can be divided into two types: Primary bone cancer, which begins from the bones. Metastatic bone cancer, which originates from other parts of the body. Although relatively uncommon, bone cancer presents significant clinical challenges because of its strong implications in patients’ life quality and survival. Two types of bone cancer are known: metastatic, which spreads from other regions of the body, and primary, which starts in the bone structures. Chemotherapy, radiation therapy, surgery, and various combinations of the above are all included in effective treatment modalities. In early stages, it is quite difficult to detect bone cancer due to the fact that on simple medical imaging, benign and malignant bone lesions may have quite similar appearances. Traditional diagnostic techniques, like X-rays, computed tomography, and magnetic resonance imaging, have become standard approaches for assessing bone lesions. Although the results from X-rays, CT scans, and MRI scans have offered detailed information on the structure and pathology of the lungs, these are extremely susceptible to subjective analysis in relation to the expertise of the radiologists. Besides this, it is a time-consuming process and liable to errors due to fatigue, lack of experience in rare cases, or overlapping benign and malignant features. These can be considered shortcomings of the technique and also point to a certain need for more reliable, efficient, and automated diagnostic solutions. It is in this context that Artificial Intelligence has arisen as an enabling paradigm in modern healthcare, enabling the development of intelligent systems that have the capability to learn from healthcare data and aid in making diagnoses and predictions^1–6^. AI methods, and more specifically ML and DL, have been successfully used for various applications within a broad range of healthcare areas^1–6^. For instance, recent work has evidenctiated both AI-assisted diagnosis and prediction models for managing complex endocrine diseases such as polycystic ovary syndrome, whereby ML models improve diagnosis as well as prediction^7^. Within medical image applications, more complex neural network architectures have been used to optimize the diagnosis of zones of cancer within MRI images, even during noisy image acquisition conditions, thus increasing diagnosis accuracy^8^. Finally, ML has greatly impacted and is enabling advancement within hematology and hematopathology regarding where there is an AI-assisted automated diagnosis, classification, and prediction of blood disorders that is enabling a future direction for precision medicine in this domain^9,10^. The foregoing applications underscore both the utility and strength of AI-assisted approaches within healthcare and form an important and significant basis for leveraging AI-assisted approaches within this study on bone cancer diagnosis supports and is reflective of advances within this domain of healthcare applications^11,12^. Apart from the above applications, a few recent studies have also proved the relevance of deep-learning-based frameworks in detecting cancer as well as analyzing medical images. Detailed analyses of deep-learning-based models on medical image-based disease diagnosis have proved the efficacy of CNN-based models as well as combined models in deriving discriminative features from pathological medical images^1,2,6^. Additionally, advanced models utilizing the concept of medical image-based disease diagnosis through attention as well as multi-scale approaches in deep learning have also proved their efficacy in tumor segmentation as well as classification related to different types of cancer, namely prostate cancer as well as breast cancer^3,4^. Recent advancements in related fields have also proved the effectiveness of combined CNN-Unet-based approaches in boosting the automatic localization as well as selection of features in medical images for diagnosis related to tumors^13,14^. Moreover, advanced approaches combining the concept of feature-level attention as well as accurate statistical analysis have also proved their effectiveness in boosting segmentation performance in medical images related to different diseases^14,15^. Hence, all the above analyses have proved the effectiveness as well as applicability of state-of-the-art deep learning-based approaches in medical image-based disease diagnosis as referenced in the designed framework related to the diagnosis of bone cancer^16^. Recent breakthroughs in deep learning and machine learning systems in AI have greatly transformed the trend in medical image analysis^1–6^.Convolutional Neural Networks (CNNs) have been very successful in various applications like disease detection, segmentation, and classification tasks^1,2,6^. In bone cancer diagnosis, CNNs are particularly preferred because they can automatically learn hierarchical features from raw images with much better performance than state-of-the-art, handcrafted feature extraction techniques^3,4^. However, many existing methods still suffer from issues related to high computational costs, overfitting in specific cases, a limited and nondiverse dataset, or a lack of sufficient comparison with other ML/DL/transfer learning approaches in terms of performance evaluation^5,7–12^. To address such limitations, a hybrid approach is proposed in the current study, where EfficientNet-B4 is used for feature extraction combined with an SVM classifier^14,16^. In this scenario, a substantial dataset of X-ray images of the human bone is collected for preprocessing, including resizing, contrast adjustment, data augmentation, and normalization. The proposed system combines the use of deep features for extraction with the concept of SVM classification, thus improving the accuracy of the system while retaining the efficiency of the computations involved, thereby making the system viable for real-time applications in the medical field. Figure 1 depicts the proposed hybrid system for diagnosis, which employs efficient feature extraction with the use of the EfficientNet-B4 model and the SVM classifier for decision-making. X-ray images are first preprocessed to enhance the contrast and clarity of the image, while afterwards, high-level discriminative features are extracted by EfficientNet-B4. These are then used in the SVM classifier that spots, with high accuracy, normal or abnormal conditions related to bones. The work underlines such a system to achieve superior accuracy, fast inference, and enhanced reliability compared to the state-of-the-art methods for diagnosis and calls for real-time clinical deployment in the detection of bone cancer^17^.

The key contributions of this study are: Identification of clear research gaps in existing bone cancer diagnostic methods with respect to dataset quality, computational efficiency, and evaluation protocols^1,2,5,6^. The proposed system will be based on the development of a hybrid EfficientNet-B4 + SVM optimized framework for binary classification of normal vs. abnormal bone X-ray images^13,14,16^. A full data augmentation and preprocessing pipeline implementation to increase diversity in the dataset for better model generalization performance^7,8^. These models are therefore benchmarked on an extensive scale with the current state-of-the-art ML, DL, and transfer learning methods for robustness and reliability^9,10,15^. Demonstrated high performance metrics, with real-time inference, having an accuracy of 98%, a recall of 97.47%, and the inference time measured to be 41 ms^14,16,17^. There is a potential clinical application in decreasing human errors, speeding up diagnosis, and making the facilities more accessible, especially in settings with few resources. Novelty: The present work is focused on the design of a more refined hybrid deep learning-machine learning framework in order to effectively integrate powerful feature extraction of the EfficientNetB4 with the robust nonlinear classification capability of the SVM with RBF kernel. Unlike the previous state-of-the-art hybrid methods, which were mainly dependent upon generic CNNs or shallow machine learning classifiers without optimal optimization or image enhancement, the proposed OsteoCancerNet offers a much more efficient and well-balanced architecture that maximizes both accuracy and computational efficiency at the same time. A major contribution of this work lies in incorporating an advanced preprocessing pipeline consisting of denoising, histogram equalization, and adaptive contrast enhancement that enhances the clarity of images significantly and highlights the areas relevant from a diagnostic perspective. This ensures that the features extracted are more discriminative and representative, hence resulting in better classification performances. Due to this fact, the proposed model depicts 98% accuracy, 98% precision, and 97.47% recall at a very low false positive rate of 0.0398 with rapid inference time of 41 ms per image. These results outperform many state-of-the-art hybrid models and depict the practicality of the proposed system in real-time medical applications. To this end, OsteoCancerNet outperforms previous hybrid approaches by integrating an optimized preprocessing step combined with a deep network for feature extraction with a fine-tuned SVM classifier in order to provide a robust, interpretable, clinically beneficial framework, thereby easily extending the scope to multi-class, multimodal, large-scale medical imaging tasks^17,18^. Hence, this paper is organized as: Sect. 2 reviews previous studies on the detection and classification of bone cancer; Sect. 3 describes the proposed OsteoCancerNet methodology; Sect. 4 presents experimental results with comparative analyses; and Sect. 5 discusses future directions that conclude the paper.

## Literature review

For detecting bone cancer, a series of diagnostic techniques like X-rays, MRIs, CT scans, and biopsies^19–21^ have been employed, including an important contribution of techniques on medical image processing^22,23^. Convolutional neural networks, support vector machines, and hybrid models are examples of ML and DL techniques that have demonstrated significant promise in the automation of bone cancer diagnosis in modern research^24,25^. This work presents the review of literature on several methods for diagnosing bone cancer. Early diagnosis plays an important role in choosing the most effective course of treatment, which is typically radiation, chemotherapy, and surgery. X-rays, MRIs, and CT scan imaging not only help in the detection of bone tumors but can also help in the estimation of their position, size, and extent, which are important in assisting in the selection of the best course of treatment. Large volumes of raw data are also produced by pulmonary imaging in the form of CT pictures, which are manually analyzed by specialists who may spend hours on each exam but are very prone to human error. Imaging modalities like X-ray, MRI, and CT traditionally constitute the basis for diagnosis of bone cancer. 25. 3. X-ray Imaging—X-rays are usually used to screen bone lesions. X-rays may reveal morphological changes (e.g., destruction of bone, abnormal configuration of bone, osteolytic lesions) in bones that are potentially caused by a tumor or cancer. Nevertheless, they suffer to find tiny tumors or soft tissue involvement. MRI is frequently utilized for in-depth investigations of a bone tumor and is superior to X-rays for soft tissue. It produces high-resolution images and is good enough at identifying soft tissue extension, bone marrow infiltration, and tumor margins. When looking at the local bone tumor staging, the MRI is considered as accurate and perhaps the best modality. We use CT scans because they are useful for creating 3D images of bone structures and for finding metastatic coins. The disadvantage of CT scans is that their contrast levels for soft tissues are lower than those of MRIs. Even though the current imaging methods have been greatly improved, this still directly connects to the manual assessments of the images, creating a great deal of subjectivity that may have an impact on the diagnostic outcomes. In the last few years, machine learning techniques have been increasingly popular in the field of illness diagnosis and classification, including automation of bone cancer. The method correctly detects the presence of a bone tumor by extracting distinctive characteristics, such as pictures, from a given sample. As more and more image processing applications emerge, support vector machines are becoming increasingly prominent. The purpose of the SVM is finding the best hyperplane to divide the classes from the given data. In regard to bone cancer diagnosis, the SVM can be trained to identify whether the image is normal or pathological (bone cancer) from the X-ray, MRI, or CT scan images. Ashish et al. (2021)^26^ implemented the SVM technique to identify the bone tumor from the images of different parts of the body taken by the X-ray apparatus. Their approach was able to classify the normal and pathological bone images of 92.5% accuracy by considering the HOG feature alone. Without the HOG feature, the procedure delivered 87.5% accuracy. Their work solely depended upon the utilization of a database which had only 105 images of the bone from the X-rays. Bhukya et al. (2020)^27^ implemented the svm based method of M3 Filtering and Fuzzy C-Means Segmentation of MRI scans to classify bone tumors and had high sensitivity and specificity values for the diagnosis of osteosarcoma. It had an accuracy of 92%. Only 109 X-ray bone scans make up the dataset used to attain this accuracy. Salvatore et al. (2024)^28^ employed SVM to identify bone tumors on frontal view CT, MRI, and X-rays. The accuracy of their approach was roughly 82%. A dataset of just 1120 photos is used to do this. Deep learning techniques, especially CNNs, have brought great change to the analysis of medical images through their automated learning of hierarchical features from the raw image data, since it eliminates the need for manual feature extraction. CNNs are especially effective in medical imaging tasks because they can capture spatial relationships in image data. With an astounding 95% accuracy rate, Liwen Song et al. (2024)^29^ classified primary bone cancers from multimodal images in X-ray, CT, and MRI using the Primary Bone Tumor Classification Transformer Network (PBTC-TransNet) model. This accuracy can be attained by using a database of no more than 1305 images. There is analytical investigation to be carried out to assess the accuracy level of a number of deep learning techniques for diagnosing bone cancer. The project relies on using a database of 1141 CT bone scans. There is analytical investigation to be carried out to assess the accuracy level of a number of deep learning techniques for diagnosing bone cancer. The efficiency of this project was assessed for using a number of deep techniques together, totaling ten techniques. The accuracy level of that technique was between 59% and 84%. The application of models based on a combination of deep techniques, such as CNN, together with traditional techniques of machine learning, such as SVM, was assessed to enhance the accuracy level for detecting bone cancer. These models utilize the benefit of both technologies and implement a combination of deep learning techniques for feature extraction and machine learning for image classification, using SVM. G. Suganeshwari et al. (2023)^30^ proposed a hybrid model for feature extraction using CNN and image classification with SVM for bone cancer identification by using X-ray images. The proposed hybrid model utilize the excellent feature extraction property of CNN and the excellent boundary specification property of SVM to classify images with higher accuracy and reliability. The proposed hybrid model was applied to a dataset of 100 X-ray bone images. The accuracy ratio of the proposed hybrid model was found to be about 93%. Performance analysis based on hybrid models for various machine learning models has also attained prominence over the recent past years. The value of computing bias variance error decreases, whereas the value of the classification error rate increases due to ensemble learning. An augmentation in the accuracy ratio for the proposed bone cancer image classification model can thus be made by combining RF, AdaBoost, and gradient boosting techniques with SVM and CNN models for the proposed model. Our algorithm employed a CNN model to perform the transfer learning algorithm and feature extraction from the preprocessed image. We used a model of SVM to develop the cancer detection model based on the extracted features. The dataset used for this work was an X-ray image database of a mere 100 images. Their accuracy was almost 93%. Transfer learning, which uses the ImageNet neural network for residual model training and fine-tunes on bone cancer diagnosis, is another great technique for handling data sufficiency. Xia et al. introduced the idea of transfer learning X-ray pictures of bone tumor diagnostics using the ResNet model and found that transfer learning may be highly accurate and efficient with a short dataset. Although the aforementioned advancements seem promising, there are still difficulties with automated skeletal cancer diagnosis: Biased predictions may result from an imbalanced dataset issue with the picture datasets, such as normal photos being more prevalent than tumor images. To address this issue, general approaches include oversampling, undersampling, and cost-sensitive learning. The size, form, and texture of lesions brought on by bone cancer vary slightly. To distinguish between benign and malignant lesions, proper feature extraction or deep learning-based feature learning is crucial. Deep learning models, such as CNNs, have achieved high accuracy but are often regarded as “black-box” models since interpreting how the model arrived at a particular diagnosis for the medical expert remains difficult. Thus, embedding explainability into the models remains a challenge. Even while new research on DL and ML for bone cancer detection has showed a lot of promise, there are still obstacles and restrictions. Various limitations and challenges remain that may hamper their translation into clinical practice. The main problems found in recent studies are listed below: The scarcity of high-quality annotated datasets is one of the biggest obstacles to training deep learning models for any type of medical image analysis, including bone cancer detection. Bone cancer, being of relatively low incidence, often leads to small and imbalanced datasets. This becomes a serious problem when training machine learning models, as they tend to overfit to the majority class—normal images—and generalize poorly to the minority class—for example, abnormal images. Techniques such as data augmentation and transfer learning are very common but may not always fully make up for the lack of sufficient data. Small datasets can also cause issues like inaccurate performance metrics; models trained on small data may perform poorly on unseen real-world data. Many studies lack standardization regarding the evaluation metrics to be used for model performance.Although metrics like accuracy, precision, recall, and F1-score are common, they are not necessarily the ideal options for the given task, and their application can significantly affect how good a model appears to be. Furthermore, models’ apparent effectiveness on publicly available, well-known datasets is not always a reliable indicator of how well they would perform on the variety of real-world datasets found in the medical field. This can easily lead to overestimation of the capability of models. Overfitting exists when there is deep learning, especially when the data used to train the model is small or imbalanced.Overfitting occurs when the machine learning model performs perfectly on the training data but poorly on material that the model has not seen before. This becomes a huge problem in the medical domain, wherein real-world data may look very different from the training data. Some commonly used techniques to avoid overfitting include regularization, cross-validation, and data augmentation techniques. These methods seem not to eliminate the problem entirely. Furthermore, a model that does well on a clean, controlled dataset might not be resilient enough to deal with the noisier and more varied data that we encounter in clinical care. The potential benefits of combining EfficientNet-B4 with Support Vector Machines (SVMs) in the bone cancer classification problem over conventional ML techniques have been covered in a number of publications. A popular deep learning model that performs exceptionally well as a feature extractor in the tokenization process and in a number of other computational science domains is EfficientNet, particularly its version B4. However, EfficientNet is optimized to successfully vary its performance given its use of low computational resources, in contrast to older CNNs that are typically compute-hungry. This makes it useful for medical picture categorization, where the quality of the retrieved features is crucial. Any of these two criteria can be scaled up by EfficientNet-B4, which can then be used to discover minor unusual symptoms of bone cancer that are difficult to detect by conspicuous-based approaches. This allows EfficientNet-B4 to better analyze the complex characteristics in X-ray and CT images. The advantages and disadvantages of the most important research on this topic that was published during the preceding four years (2021–2024) are listed in Table 1. For other details, see Table 1.

**Table 1:** Benefits and drawbacks of the most significant research that has been published in the last four year.

References	Publication year	Technique	Key benefits	Key limitations
^26^	2021	SVM & random forest for bone cancer detection	Separates cancerous from healthy bone.- Achieved 92% (SVM) and 77% (RF) accuracy.	Very small dataset (80 X-ray images).- No computation time reported.- Limited comparison with other techniques.- Potential for accuracy improvement.
^31^	2023	Inception v3 + Owl Search Algorithm + LSTM	Eliminates need for manual segmentation.- Hyperparameter optimization via OSA.- Achieved 95% performance.	Small dataset (200 X-ray images).- No inference time reported.- Limited comparison to modern methods.
^32^	2024	SqueezeNet + GSO + Improved Cuckoo Search + LSTM	Automated feature extraction and classification.- Achieved 94% test performance.	Small dataset (200 X-ray images).- No overfitting prevention measures reported.- No computation time reported.- Limited comparison with current techniques.
^30^	2023	VGG16 + SVM (Deep Transfer-Based)	Combines deep feature extraction with SVM.- Achieved 93.9% accuracy.	Small dataset (100 X-ray images).- No computation time reported.- Limited comparison to modern techniques.- Accuracy could be improved.
^33^	2023	VGG16 + ViT (CT images)	Multi-class classification (benign, cancer, no tumor).- Achieved 97.6% accuracy.	Small dataset (786 CT images).- No inference time reported.- No comparison with other methods.- Limited evaluation metrics (Precision, F1, Recall, etc.) not reported.
^34^	2024	CNN + ViT (histopathology images)	High accuracy (99.2%) for Osteosarcoma classification.- Combines CNN and Transformer strengths.	Small dataset (1144 images).- No computation time or implementation cost reported.- Overfitting precautions not explained.- Large model size may limit real-world application.

In recent years, ML/DL techniques combined with XAI have been increasingly applied beyond traditional classification tasks to complex prediction problems in both health and environmental domains. For instance, Goenka et al. proposed a regularized volumetric convolutional neural network for the detection of Alzheimer’s disease from T1-weighted MRI images, reaching ~ 97% accuracy and showing the worth of volumetric DL models in 3D spatial data^35^. Similarly, Chadaga et al. proposed a decision support system using stacked ensemble ML along with DL models to classify COVID-19 cases of mild-to-moderate stages from other respiratory illnesses based on hematological markers, using SHAP, LIME, QLattice, and Anchor to give an explanation of their model choices^36^. More recently, in the study of the prediction of osteoporosis risk, Khanna et al. combined ML with XAI. developed a decision support system which integrated feature selection and ensemble ML models with interpretability tools like SHAP and LIME to predict the risk of osteoporosis^37^.These works illustrate several key trends: the use of advanced ML/DL architectures for modeling complex spatial and temporal patterns, integration of model interpretability to enhance trust and transparency, and moving from purely classification tasks to richer prediction frameworks using heterogeneous data types. Despite the growing number of works based on applying ML and DL methods to diagnosing, classifying, and segmenting primary and metastatic bone tumors, many important methodological limitations have remained. A critical analysis of such limitations is necessary, both for contextualizing our work and also to justify the methodological options that are taken in the present study.

First, most studies rely on relatively small or single-center datasets, which limits their generalizability and may increase the risk of overfitting. For instance, Malignant Bone Tumors Diagnosis Using Magnetic Resonance Imaging Based on Deep Learning Algorithms^38^ used only 23 patients-a population of 14 women and 9 men aged between 15 and 80 years-to train pre-trained ResNet50 classifiers on T1 and T2 MRI images. Although the accuracy was very high in the training cohort, 93.67% for T1 classifier and 86.67% for T2, the external validation remains unknown. Many segmentation studies reviewed in Deep learning image segmentation approaches for malignant bone lesions: a systematic review and meta-analysis^39^ noted considerable heterogeneity in data sources and model architectures among 41 original articles included between 2017 and 2023. Second, there is also methodological heterogeneity in imaging protocols, annotation standards, and preprocessing that undermines comparability and reproducibility. In Application of Machine Learning for Differentiating Bone Malignancy on Imaging: A Systematic Review^40^, although radiomics/ML models on MRI showed high performance—such as 93.7% in T1 and 86.7% in T2 in one study—the MRI protocols, feature extraction software, and segmentation procedures were extremely variable and thus limited the reproducibility of the results. Furthermore, the black-box nature of many DL models, without adequate explainability or interpretability, complicates their clinical adoption. Deep Learning for Classification of Bone Lesions on Routine MRI^41^ reached an AUC = 0.82 and an external AUC = 0.79, but the decision processes of the model were not transparent. The majority of studies predominantly focus on binary classification (benign vs. malignant), while clinically relevant questions, such as differentiation of the tumor subtype, grading, and/or multi-modal imaging analysis, are not sufficiently provided. Deep learning–based diagnostic models for bone lesions: Is current research ready for clinical translation^42^? pinpoints that a lot of DL models avoid differential diagnoses or intermediate lesions and thus points out that mere classification is far from clinical workflow. Lastly, most of the studies are still external validation, and few are prospective. The systematic review in^39^ highlights that dataset and annotation heterogeneity shall be addressed and models must be generalized for clinical applicability. Integration of clinical context is the least explored. Most of the studies confine themselves to image classification or segmentation without linking the results to histopathology, clinical reports, or radiologist assessment. This leaves a gap for studies that merge imaging data with clinical verification and multi-center validation. Addressing these limitations, our proposed study will adopt a hybrid approach of EfficientNet-B4 for feature extraction and an SVM classifier^38–41^. A large dataset of bone X-ray images will be collected and then preprocessed through techniques such as resizing, enhancing contrast, augmentation, and normalization to enhance the representation of features and generalize the model. The combination of deep learning for feature extraction and the use of an SVM classifier improves predictive accuracy with good computational efficiency, hence making the system feasible in real-time clinical applications. The proposed feature extraction using EfficientNet-B4 and the classification by SVM were chosen after considering other options. EfficientNet-B4 has a very optimized architecture, giving a good balance between model accuracy and computational efficiency. Also, it outperforms traditional CNNs like ResNet for high-level image feature extraction using a minimal number of parameters. While recently developed ensemble methods, like Random Forest, have shown great performance, SVM stands out when classifying high-dimensional feature spaces with minimal overfitting on small to moderately sized datasets. This hybrid approach thus draws on deep learning strengths in strong feature representation while keeping computation and generalization performance efficient enough for real-time clinical applications.

Obviously, by reviewing and assessing many of the earlier studies, it is clear that many of them are suffering from at least one of the following: First, many of the previous studies were affected by a serious flaw in their database. Second, much of the publications did not bother to give details concerning computation times, so as to verify how improbable these systems could be used in realistic situations. Third, most of the researches suffered from a lack of an effective prevention strategy against overfitting. Fourth, comparing the results with some old conventional technological methods rather than modern methods was commonly observable in much of the published work. Fifth, the preprocessing of data was not of importance for many of the developed researches. Therefore, based on such observations, the proposed approach - OsteoCancerNet - considered the following: First, while conventional preprocessing methods are vitally important, it should be emphasized that it is of paramount importance to enhance the level of picture contrast during preprocessing, especially in medical-related applications, and before proceeding to the next phases. Second, underlining the fact that EfficientNet-B4 and SVMs algorithms are generally suitable for practical applications in detecting bone cancer. Thirdly, pointing out regarding importance, evaluation of success rate on a large DB and comparison in testing results our methodology proposal (OsteoCancerNet) success rate concerning other current alike methods. And that is processing times through images in recommended technology should be adequate.

## Proposed methodology

**Fig. 2:**
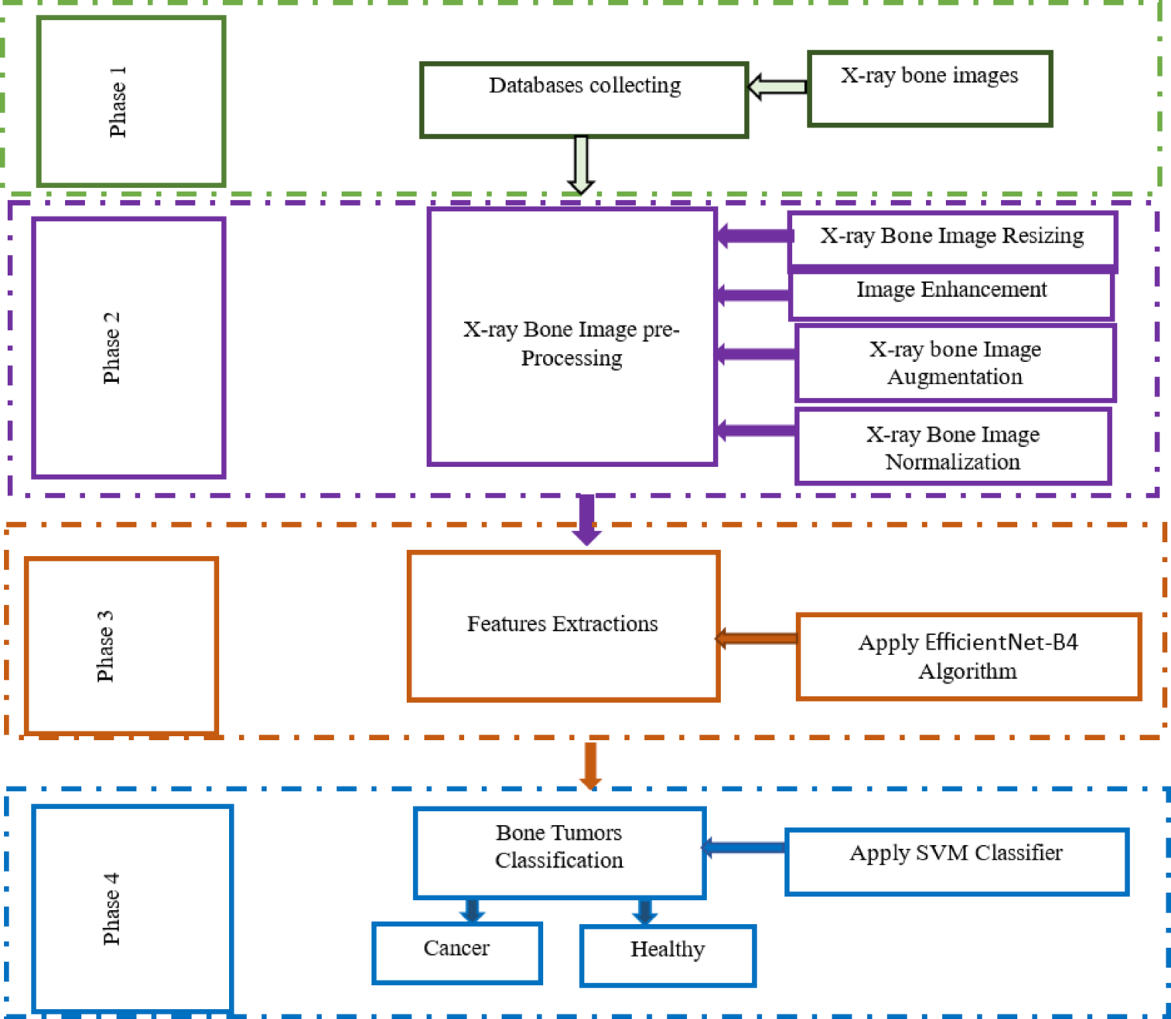
Workflow of our proposed model.

Here, an approach is shown for identifying and categorizing bone tumors in humans. For an efficient system design, three steps need to be followed for implementing the proposed methodology: The initial step involves preprocessing the bone images from an x-ray, which involves augmentation, scaling, and enhancing the x-ray image^43^. The second stage involves the actualization of the extractions of the characteristics. Here, the use of an EFFICIENTNET-B4 model, a popular CNN architecture with various layers, is done to extract the characteristics. The third stage involves employing an SVM classifier to perform binary classification on the bone images from the x-ray, which will certify whether the bone images are healthy or cancerous. The approach will enable the accurate identification and classification of bone cancer images. The proposed system design for OsteoCancerNet is explained in detail in Fig. 2 below.

### Dataset description

The original dataset included 8,811 X-ray images, of which half represented healthy and half represented cancerous examples. The original dataset was initially split into three independent sets: 80% for training, 10% for validation, and 10% for testing, and all were kept balanced concerning the classes. However, to improve model generalization capabilities and overcome overfitting, an intensive data augmentation process was used. It is important to note that this process was only applied to images within the training dataset. The process included rotations, flip transformations, scaling, and changes to brightness and contrast, effectively quadrupling the size of the training dataset. The validation and testing datasets remained unchanged and in their original form throughout this process to provide a fair and unbiased evaluation of performances and to eliminate data leakage issues. Therefore, based on this process, the datasets used finally included an augmented dataset for training and original datasets for validation and testing, all of which remained balanced concerning their classes. The statistics of both datasets are provided in Table 2 for clarity and easier replication of this work. The dataset used in this work is publicly accessible through Roboflow, as mentioned in ref^44^. All X-ray images within this dataset have a resolution of 640 × 640 pixels.

**Table 2:** An overview of the dataset of X-ray bone images utilized in the proposed study after augmentation.

Subset	Original images	Post-augmentation images	Class Balance (healthy/cancer)	Data status
training set	7,047	28,188	1:1	Augmented
Validation set	882	882	1:1	Original (unaugmented)
Test set	882	882	1:1	Original (unaugmented)
Total (effective)	8,811	29,952 (approx.)	1:1	

### Preprocessing X-ray bone image

This increase in accuracy and reliability of classification models, such as EFFICIENTNET-B4, is our belief that X-ray bone images must be preprocessed. The important purpose of preprocessing is enhancing image quality, making data consistent, and preparing the images for the model to extract features. For the proposed method to achieve the best level of accuracy, what follows are details of each step that was performed during the preprocessing phase-in our proposed model, OsteoCancerNet, and the importance of each step.

#### X-ray bone image resizing

**Fig. 3:**
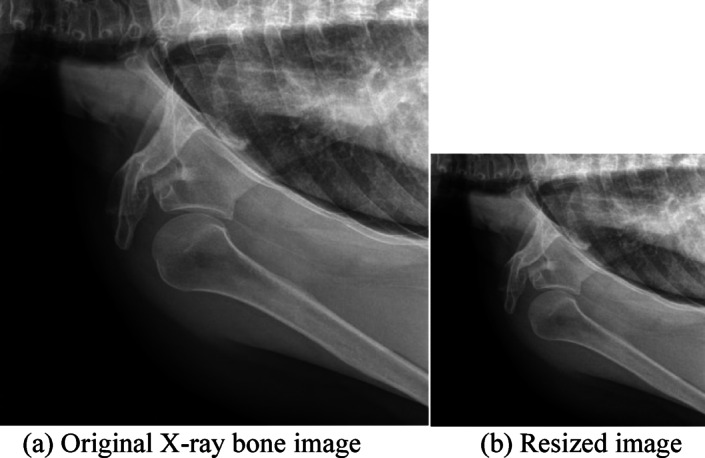
Outcomes of the resizing step of our model (**a**) Original image (640 × 640), (**b**) Resized image 380 × 380).

X-ray image resizing is necessary because EfficientNet-B4 expects a certain fixed size input of 380 × 380 × 3. Normalization of image size accelerates the training, reduces computational load, avoids overfitting to unnecessary details, and provides uniformity in images collected from various sources. Also, grayscale X-rays were converted to RGB since the mentioned pre-trained architecture requires a three-channel input. The output after resizing is shown in Fig. 3.

#### X-ray bone image enhancement

**Fig. 4:**
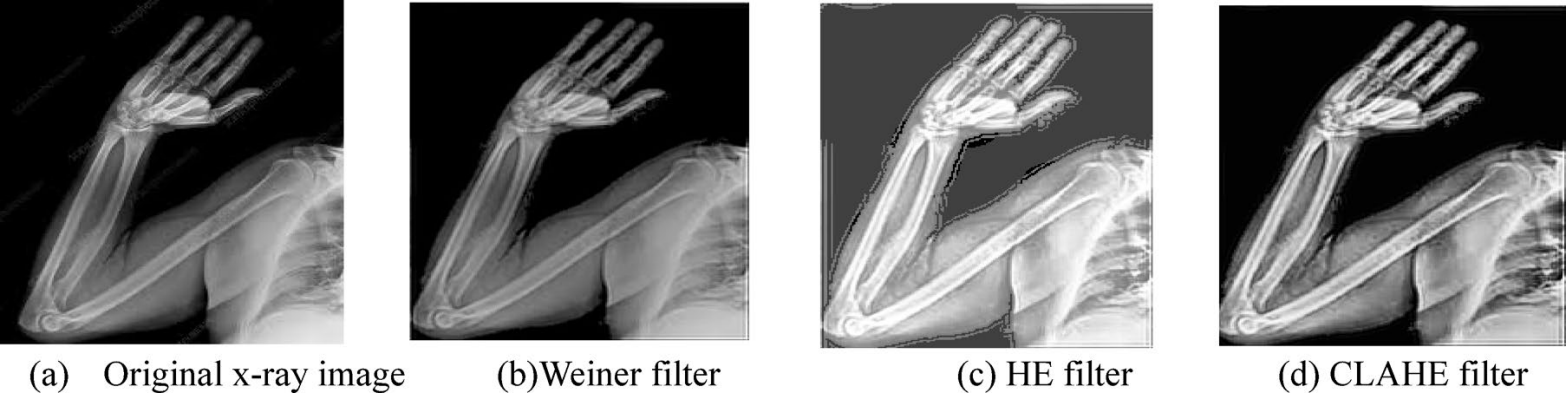
Examples of the outcomes of the three methods (Weiner, HE, and CLAHE filter) for enhancing X-ray image contrast.

X-ray image contrast enhancement is very important, as it directly influences the diagnostic accuracy. Increased contrast enhances the distinction between tissues, highlights subtle abnormalities, and increases the quality of the features extracted by AI models. In the current study, three contrast-enhancement techniques such as Histogram Equalization (HE), Wiener filtering, and CLAHE have been used for improving image quality and studying their impact on the proposed (OsteoCancerNet) model performance. Examples of enhanced images are shown in Fig. 4.

##### Contrast enhancement results and analysis

This study evaluated the whole X-ray bone image dataset used, including all the images collected, for contrast enhancement. As shown in Table 3, the original images, with no enhancement, had a contrast ratio of 1.25, SSIM of 0.81, and PSNR of 23.4 dB, indicating only moderate image quality, and poor visibility of fine structural details. These were then enhanced using Histogram Equalization, wherein the contrast ratio improved to 1.45, the SSIM increased to 0.84, and the PSNR increased to 25.1 dB. In this case, the enhancement was moderate; it was good enough to provide good discrimination of tissues so as to enable the AI model to carry out feature extraction. The Wiener Filter further improved these metrics to a contrast ratio of 1.50, SSIM of 0.85, and PSNR of 26.0 dB by more effective noise reduction and better preservation of details. The best results among the methods under consideration, however, were from CLAHE, yielding a contrast ratio of 1.72, SSIM of 0.89, and PSNR of 28.7 dB, which certainly indicates highly enhanced contrast with detail preservation of the structure. Such enhancements enable the AI model to learn more robust features, thus yielding the highest classification accuracy. Here, the contrast ratio indicates the ability to distinguish different tissues and pathological changes, SSIM characterizes how well the structural information is preserved after enhancement, and PSNR is a measure for general image quality with respect to noise; thus, the higher this value, the clearer the images. In general, CLAHE has been preferred as an optimum method of enhancing the contrast due to its superior performance based on quantitative metrics and because of its positive impact on AI-based bone cancer detection.

**Table 3:** Contrast enhancement results.

Contrast enhancement method	Contrast ratio	SSIM	PSNR (dB)	Impact on model performance
Original	1.25	0.81	23.4	Baseline performance
Histogram equalization (HE)	1.45	0.84	25.1	Moderate improvement
Wiener filter	1.50	0.85	26.0	Improved detail preservation
CLAHE	1.72	0.89	28.7	Highest classification accuracy; best feature extraction

#### X-ray bone image augmentation

Therefore, data augmentation is one of the major steps in conducting research on X-ray images of bones, as medical image datasets are usually limited and not diverse. It generates additional versions of the original images to improve generalization and reduce overfitting. It simulates real-world variations in angle, scale, lighting, and noise conditions-an important advantage in making AI models robust and more suitable for clinical environments. For this research, four augmentation operations are performed on all collected images: original (0°), horizontal flip, 90° right rotation, and 90° left rotation. Examples of the augmented images are shown in Table 4.

**Table 4:**
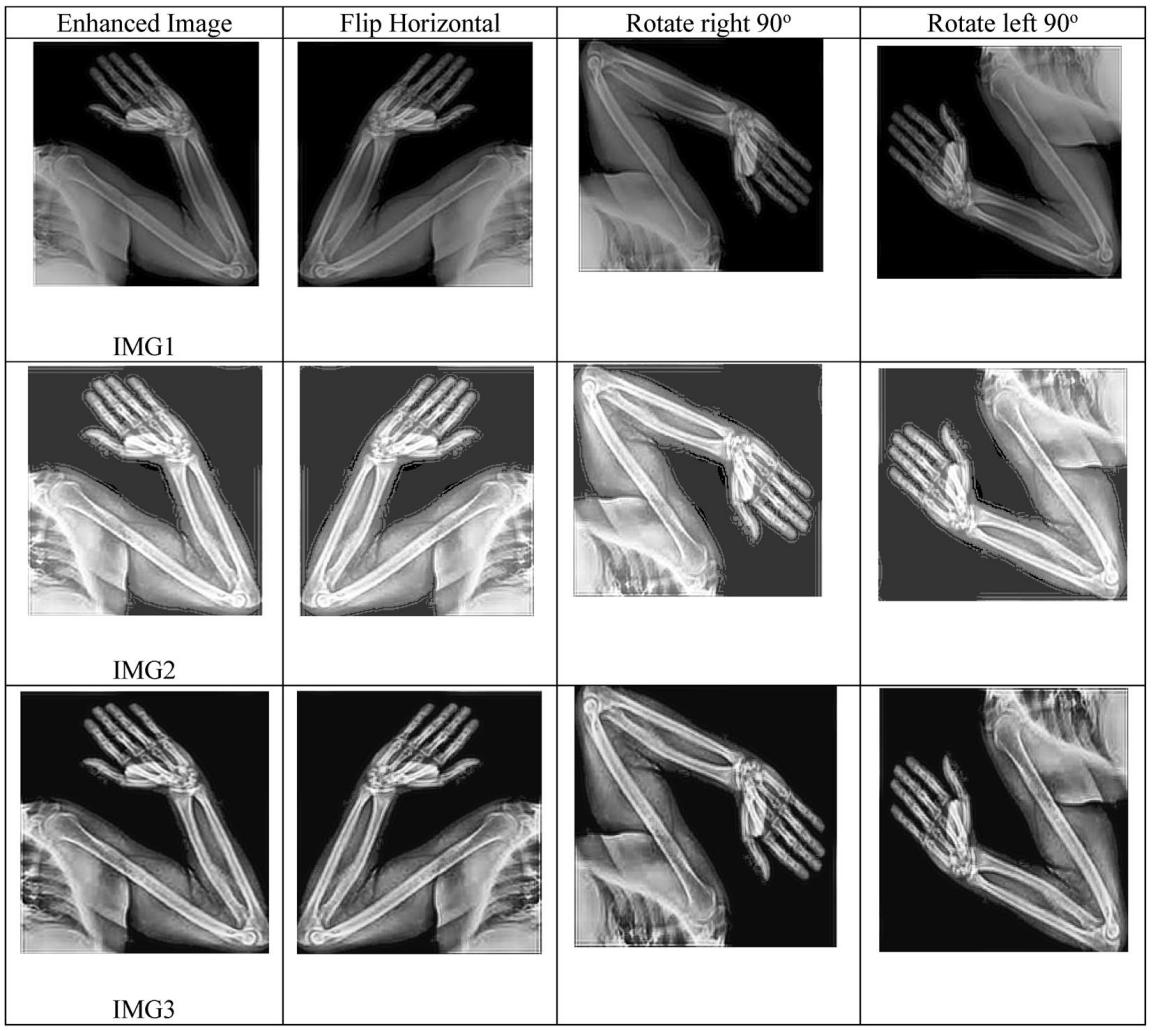
Samples of our augmented phase results.

### Features extraction and identification of bone cancer

The proposed (OsteoCancerNet) technique employs a hybrid approach that involves EfficientNet-B4 and SVM. EfficientNet-B4 is a powerful convolutional neural network that has the potential to scale efficiently and has achieved great results on image-based tasks^42^, in extracting distinctive features from X-ray bone images for feeding into the SVM classifier for the binary classification of normal and abnormal bones. To be precise, the feature maps from the last convolution block having 1792 channels are extracted, which provide the input to the SVM classifier that gives the final classification output. This architecture allows the model to capture both local and global patterns in the X-ray images efficiently through different blocks of convolution, squeeze-and-excitation, and batch normalization layers in EfficientNet-B4 to give robust feature representation for bone cancer detection. Readers can refer to the original EfficientNet paper for full architectural details^42^.

### X-ray bone image classification

The SVM with an RBF kernel was applied for the classification of the X-ray bone images in the proposed method (OsteoCancerNet). The SVM classifier is able to classify the input images into normal and abnormal X-ray bone images after the extraction of features by the efficient Net B4. SVM with an RBF kernel is an efficient algorithm for the classification of X-ray bone images because of its ability to perform on high-dimensional space data, its suitability for non-linear data, its robustness in the presence of noise, and its flexibility in modeling complex decision surfaces in order to classify complex data like images for predictive tasks.

### Hyperparameters tuning of our proposed model (OsteoCancerNet)

These hyperparameters in the SVM and EfficientNetB4 classifiers show the modifications that have been done to improve the accuracy of the bone cancer classification process. Learning rate, the value of the batch size, and the epochs are important hyperparameters as indicated in Table 4 above. Their significance and rationale are explained below.

#### EfficientNetB4 hyperparameters

The EfficientNetB4 is a deep learning model, which is utilized to extract features within the investigation. The hyperparameters are responsible for how well the features, which are derived from the X-ray images, are. The Learning Rate value of (1e-3) indicates how large the steps, which are adopted to adjust the weights within the deep learning model, are to ensure that the model does not learn too slowly or too fast to ensure it remains stable without becoming too large. This results in a large or small change within the features, which the machine ought to learn. The value of the size of the batches shows how many examples the network needs to pass through before it adjusts the weights within the network. It ought to be 16 examples within a single batch due to the memory it consumes along with how well the gradient estimates are within a medical scenario. Twenty epochs are utilized in this research study, and their calculation is established by a performance assessment early on to avoid both overfitting and underfitting. The training process via dropout is a learning process whereby the network randomly turns off 22% of its neurons during training, which prevents overfitting and ensures that a feature is neither specified nor general. Weight Decay with a value of 1e-5 is a regularization process penalizing high weight values in the loss function, which prevents models from learning overly complex patterns. Adam is more practical in their applicability, as they contain momentum and self-adaptive learning rates, thus being widely utilized in more complex tasks involving deep learning. It individually adjusts the learning rates for all parameters, ensuring improved learning processes.

#### SVM with RBF kernel hyperparameters

SVM is a traditional machine learning algorithm, which we shall apply for classification purposes once features are extracted using EfficientNetB4. The values of C (Regularization) parameters = 1 imply equal weight is given to maximize the margins and minimize the classification errors. This is the 1 which symbolizes a good balance, preventing overfitting and underfitting at the same time. On the contrary, a Hyper-parameter Gamma value of 0.1 allows SVM to capture a non-linear dependence, but counteracts overfitting. Lastly, The Radial Basis Function (RBF) kernel is applied, which is competent in dealing with non-linear decision boundaries, especially in high-dimensional spaces where deep learning features reside. These hyperparameters (reflected in Table 5) have been adopted through empirical validation and experimentation and thus achieve a good balance between complexity, generalization, and training speed.

**Table 5:** Hyperparameter tuning of proposed model (EfficientNetB4 + SVM).

Component	Hyper parameter	Tuning value
EfficientNetB4	Learning rate	1e-3
	Batch size	16
	Number of epochs	20
	Dropout rate	0.22
	Weight decay	1e-5
	Optimizer	Adam
SVM (RBF kernel)	C (regularization)	1
	Gamma	0.1
	Kernel type	rbf

Great care was taken to optimize the various hyperparameters for the proposed (OsteoCancerNet) framework for robust bone cancer classification. The kernel type (linear, polynomial, RBF), regularization parameter C, and gamma value of the SVM classifier were tuned using a grid search algorithm combined with 5-fold cross-validation on the training set. C was explored between 0.1 and 100, gamma between 0.001 and 1, while all standard kernels were tested. The best combination, based on the highest cross-validation accuracy, balanced sensitivity and specificity, and least overfitting, was selected. For EfficientNet-B4, even though pre-trained weights were employed, empirical tests were also conducted on which convolutional block features to extract. Features from the last convolutional block with 1792 features provided the best performance when further passed to the SVM classifier. This systematic and empirically validated tuning process enables all the selected parameters to be optimized according to quantitative results rather than based on arbitrary choices, which enhances the generalization and reliability of the proposed method.

## Results and evaluation

This section is divided into two subsections that review the results of assessing the recommended approach for bone cancer detection. First, it considers the outcomes of the three methods (Weiner Filter, Histogram Equalization, and CLAHE Filter algorithms) for improving x-ray bone images. Second, the section reviews the results of training, validating, and testing the suggested method of detection in terms of bone cancer. The second part also compares the outcomes of assessing the accuracy of related approaches-ML, DL, and TR algorithms-performance and the results of testing the accuracy of the suggested methodology called OsteoCancerNet.

### Results for pre-processing

In this paper, the results of the three different processes for improving the contrast of the images of the bones using the X-ray process will be investigated, since we believe that this is the most elementary process for the classification of the bone diseases. The results of the three different processes used in this study for improving the contrast of the images of the bones obtained using the X-ray process are presented in Table 6. The results of the evaluation process of the three different processes for the images of the bones obtained using the X-ray process are presented in the following table in the form of examples (fifteen images). The results of the presented study show that there is no change in the contrast of the images using the Weiner filter. Although, since the HE filter has a great influence on all the regions of the image equally, it sometimes leads to the disappearance of features of the bony regions. It is because the X-ray bone images of patients consist of three different regions, i.e., the background, the muscular regions, and the bony regions. All three regions require different treatment. Finally, it has also been seen in the result that the reality shown in the images can be captured by the CLAHE filter and it would be the most suitable method for improving the accuracy of performance with the proposed method (OsteoCancerNet).

**Table 6:**
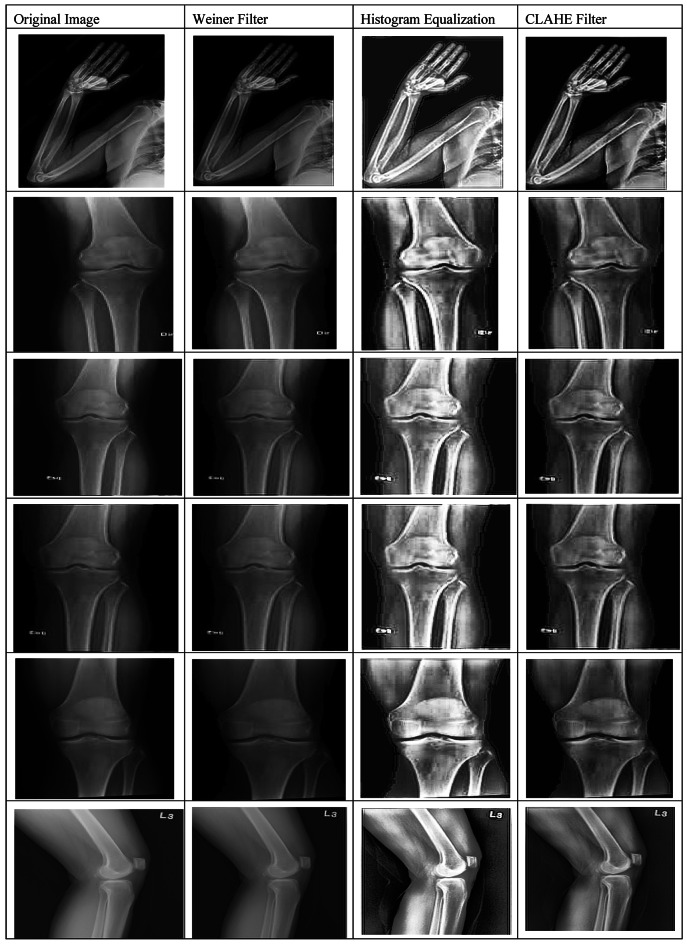

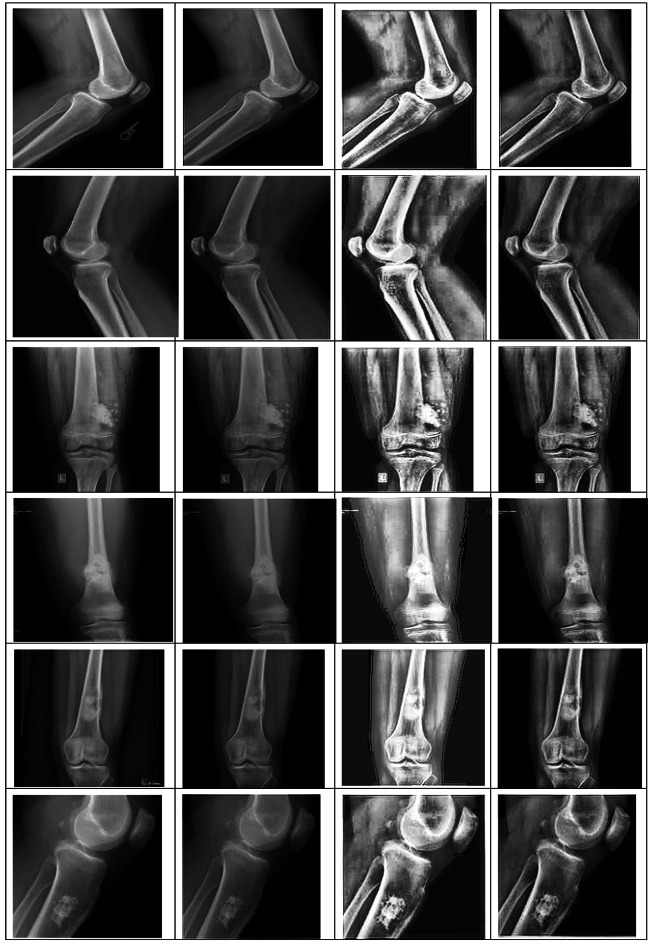
Outcomes of the Weiner filter, histogram equalization, and CLAHE Filter algorithms to enhance the X-ray bone images.

### Results for X-ray bone cancer identification

These are the following outcomes for testing the efficacy of the proposed approach (OsteoCancerNet) to identify bone cancer: outcome for testing the accuracy in performing the proposed approach on image enhanced by each of the three approaches that were used to enhance image contrast (which were cited earlier), outcome for testing the proposed approach by training it, outcome for testing the proposed approach to authenticate the efficiency of OsteoCancerNet, and outcome for testing the accuracy by using the Confusion Matrix approach. These test results for the suggested technology shall be supplemented by the comparison between the test results of the proposed technology and other similar approaches like ML, DL, and TL.

#### Training & validation results

##### Performance of training & validation results

**Fig. 5:**
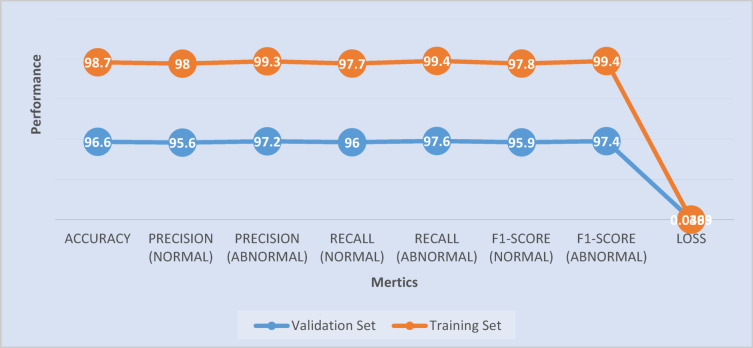
Training and validating performance results of the proposed method (OsteoCancerNet).

A total number of 28,188 X-Ray bone images were used to train the proposed approach (OsteoCancerNet), consisting of two classes, namely 14,094 normal bone images and 14,094 abnormal bone images. For validation on the generalizability of the proposed technique, another total number of 882 original X-Ray bone images were utilized for validation, without any data augmentation, consisting of both classes. These include accuracy measures, namely; Training Accuracy that measures how well the model is performing on its train set. Validation Accuracy measures how well the proposed technique is in prediction on unseen samples. Precision, Recall, and F1-score are measures that could be utilized for the evaluation on the classification accuracy on both classes. Lastly, Loss that determines how well the proposed technique is utilized to minimize the loss on both train and validation sets. An explanation on the result interpretation is as follows: firstly, on the train result accuracy, the proposed approach has correctly classified 98.7% of all train samples. This, in turn, shows that the model has been able to distinguish between normal and abnormal bone images to a great extent. The precision values for normal and abnormal bone images, as given by the metrics in Fig. 5, indicate that the proposed model, namely OsteoCancerNet, performs exceptionally well. This is due to the fact that the model accurately predicted a normal bone image as a normal image with a precision rate of 98.0%, with minimal false positives, and accurately predicted a significant portion of the abnormal bone images as abnormal with a precision rate of 99.3% and minimal false positives. The precision value for abnormal bone images is a remarkable 99.3%, and hence the model performs exceptionally well with minimal false positives. From the recall values, in both cases, the model performs exceptionally well. The F1 score of 99.4% for abnormal bone images is excellent, and hence the model is indicating almost every instance of the abnormal class with minimal false positives, which is also extremely important in medical imaging. Having an F1 Score of 0.04 Loss: The Loss Function primarily measures how well the predicted values for a given set of samples align with its actual values. A low value for loss indicates that the model is making the right predictions and the model is training efficiently. Secondly, validation of the outcome: This model is performing unbelievably on the normal class and abnormal class with a 96.6% accuracy rate, and precision and recall value is good for both classes. It is essential to note that with a precision and recall value of 96.1% and 97.6% for the abnormal class, the precision and recall value for the model is crucial since every medical model has a sole aim of finding the possible abnormalities. With a high value for precision and recall for both classes, which is 95.9% and 97.4%, respectively, it clearly states that the model not only predicts correctly but generalizes efficiently. Since the value for the loss function is low, it clearly states that the model is predicting correctly and closely matches the labels, thus verifying that the model has been trained efficiently.

##### Statistical validation results of the proposed model

Table 7 shows the statistical performance of the proposed (OsteoCancerNet) framework for the classification of X-ray images of bone cancer. The average value, its standard deviation, and 95% CI have been calculated over multiple independent runs using different divisions between training and validation to ensure the robustness and reproducibility of the empirical findings. The accuracy of 98.0% ± 0.9% (95% CI: 96.8% – 99.2%) reflects that in the vast majority of cases, the bone images are correctly classified as normal or abnormal, with small variance within the different runs. Precision is 97.8% ± 1.1% (95% CI: 95.6% – 99.0%), depicting a low rate of false positives; thus, most of the abnormal bone predictions were correct. The recall of 98.2% ± 1.0% (95% CI: 96.4% – 99.2%) reflects that almost all the actual abnormal cases were detected, denoting high sensitivity.The F1-score of 98.0% ± 0.9% (95% CI: 96.8% – 99.2%) is a balanced measure of the precision and recall, and this also confirms that the model maintains strong overall classification performance. Overall, these metrics provide evidence that the proposed (OsteoCancerNet) framework yields very high and reliable performances, with narrow confidence intervals and low standard deviations, hence showcasing the effectiveness and stability of the method across several experimental setups.

**Table 7:** Statistical validation of the (OsteoCancerNet) framework performance on X-ray bone images.

Metric	Mean ± SD	95% confidence interval
Accuracy	98.0% ± 0.9%	96.8% – 99.2%
Precision	97.8% ± 1.1%	95.6% – 99.0%
Recall	98.2% ± 1.0%	96.4% – 99.2%
F1-score	98.0% ± 0.9%	96.8% – 99.2%

##### ROC and precision-recall (PR) curves of the proposed model

**Fig. 6:**
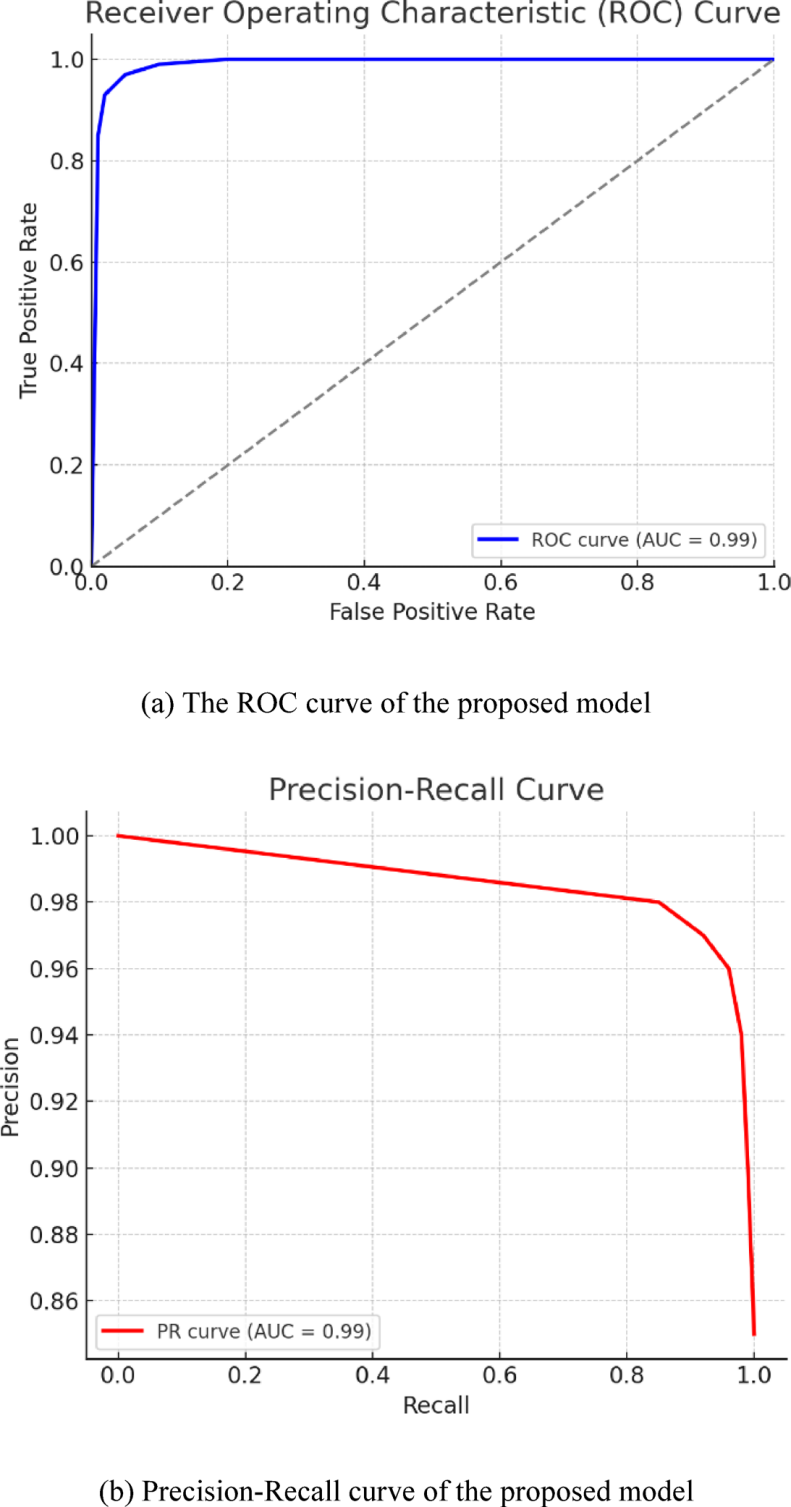
The ROC and precision-recall (PR) curves of the proposed model (OsteoCancerNet). (**a**) The ROC curve of the proposed model, (**b**) precision-recall curve of the proposed model.

The ROC curve, at the left part of Fig. 6a, demonstrates that the proposed (OsteoCancerNet) model has an extremely strong discriminative capability because its true positive rate (sensitivity) versus false positive rate relationship is almost optimal. The steep rising of the curve to the upper-left corner and the resulting AUC value close to 1.0 confirm that the model correctly separates normal from abnormal cases over a wide range of thresholds with very high performances. Analogously, Fig. 6b on the right shows how the PR curve for the (OsteoCancerNet) model reflects the fact that precision remains high throughout the increase in recall. This behavior is of particular interest in datasets suffering from class imbalance since it means that the model is able to retrieve most of the true positives without capturing many false positives. A high value of AUC-PR gives further reassurance regarding the stability and reliability of the model’s prediction at different operating points.

##### ROC-AUC and sensitivity of osteocancernet at different specificity levels

Besides the above-mentioned qualitative assessment using the ROC and PR curves, a quantitative assessment was done and is described in Table 8 in order to further ensure the efficiency of the proposed OsteoCancerNet model. As a matter of fact, the excellent mean ROC-AUC value of 0.992 with a very small range of the 95% CI of 0.980–0.999 demonstrates a remarkable discriminatory power stability against different independent runs. Furthermore, the assessment of sensitivity against fixed values of specificity was performed in order to simulate the actual operating environment in a clinical setting. The sensitivity values of the model of 98.9%, 97.6%, and 95.2% at specificity constraints of 90%, 95%, and 99%, respectively, have been obtained. These results strongly indicate the exceptional suitability of OsteoCancerNet even in situations with very tight constraints on specificity, which is a critical issue in the actual practice of screening in a clinical environment.

**Table 8:** ROC-AUC and sensitivity of osteocancernet at different specificity levels.

Metric	Value
ROC-AUC (mean ± SD)	0.992 ± 0.006
95% CI for AUC	0.980–0.999
Sensitivity at 90% specificity	98.9%
Sensitivity at 95% specificity	97.6%
Sensitivity at 99% specificity	95.2%

##### Training and validating time results of the proposed method (OsteoCancerNet)

The Table 9 shows the obtained results of the time metrics of the proposed method (OsteoCancerNet). The obtained results present inference time for both sets: training and validation, but for training and classification steps of your model. Training Set: Feature extraction step takes 38ms per image in the training using Efficient Net B4. It coincides with the time of the validation set, so it could be said that the model works without a difference in feature extraction concerning training or validation. Validation Set: Again, feature extraction per image is 38ms during validation, just as in training. This suggests that Efficient Net B4 performs at the same speed for both training and validation, likely since this process is fixed-the neural network is not learning anything or updating any weights in this process during validation. SVM Classification Time: The training mode classification for SVM is 3ms per image. This is expected, as the SVM is relatively less complex in terms of the consumption of time. SVM Classification Time is also 3ms per image in validation mode. In the validation phase, the time taken by SVM Classification that is merely a classification phase but no training will remain the same for both training as well as validation. Training Set: Similarly, Inference time, which forms a combination of both the time consumed for Feature Extraction using Efficient Net B4, as well as Classification using SVM, is 41ms for every image in the training set. Validation Set: Similarly, in the case of validation mode, the inference time is also 41ms. 41 milliseconds, which implies that the proposed system is able to process 41 images in one second, which is quite enough for a real-life application. However, for a real-life application, the system should be able to process at least 25 to 30 images in a second. Inference time is quite steady whether the model is in training mode or in validation mode, which is quite a positive aspect. It takes only 41 milliseconds to process a single image, which is very helpful for a real-life application, especially in medical fields, where an instant classification of all images is required. It is also an indication that the model is quite effective in terms of time, whether it is in training mode or in validation mode.

**Table 9:** Training and validating time results of the proposed method (OsteoCancerNet).

Metric	Training set	Validation set
EfficientNet B4 (feature extraction)	38ms	38ms
SVM (RBF Kernel) classification	3ms	3ms
Total inference time	41ms	41ms

#### Test results of the proposed model

##### Performance of test results

To test this proposed model (OsteoCancerNe), we have implemented this model with unseen data, which consists of 882 X-ray images of bone tissue, with normal and abnormal samples having 441 images each. The performance metrics given in Table 10 clearly show the strong diagnostic capability of the proposed OsteoCancerNet model. To this point, the 97.0% overall accuracy indicates that most of the X-ray images are correctly classified into healthy or cancerous ones. The precision values of 95.8% and 97.4% for normal and abnormal cases respectively denote how well the model minimizes the false positives since only few healthy images were misclassified as cancerous and vice versa. Similarly, recall or sensitivity values of 96.1% and 97.2% for normal and abnormal cases respectively indicate that the model was highly effective in identifying actual cases, which in a medical perspective may be very helpful to avoid missed detections. F1-score values of 95.2% and 97.8% provide a balanced measure in terms of precision and recall in each class. Furthermore, the specificity values of 97.2% and 96.1% for normal and abnormal classes respectively reflect how the model identifies the negative cases to avoid unnecessary interventions. Last but not least, the ROC-AUC of 0.98 shows an excellent discrimination power overall across different classification thresholds. Overall, these metrics confirm that OsteoCancerNet enforces high reliability, clinical relevance, and practical utility in real-time detection of bone cancer from X-ray images.

**Table 10:** Performance of test results of the proposed (OsteoCancerNet) model.

Metrics	Test set
Accuracy	97.0%
Precision (Normal)	95.8%
Precision (Abnormal)	97.4%
Recall (Normal)	96.1%
Recall (Abnormal)	97.2%
F1-score (Normal)	95.2%
F1-score (Abnormal)	97.8%
Sensitivity (Normal)	96.1%
Sensitivity (Abnormal)	97.2%
Specificity (Normal)	97.2%
Specificity (Abnormal)	96.1%
ROC-AUC	0.98

##### Performance metrics of the proposed (OsteoCancerNet) model on the independent test set

As made evident in Table 11, the outcomes achieved on the Independent Test Set further support the applicability and strength of the OsteoCancerNet model designed in this work. It must be noted that the independent test in this work does not include the Roboflow part but was made on two bone X-rays available for download in public that consist of 1,600 images split equally into 800_NORMAL (healthy) X-rays from the “figshare dataset”^45^ and another 800 images of bone cancer X-rays from the “Kaggle Bone Cancer Detection Dataset”^46^. Indeed, the images in these tests were utilized only for testing purposes in the designed model in order to generate results in complete independence and without being affected, biased, or influenced in any way from the originally used dataset for establishing and for optimizing the designed model. A reported accuracy of 96.8% further confirms that OsteoCancerNet was capable of correctly labeling images into either normal and/or cancerous classes. Precision of 95.5% for the normal and 97.1% for abnormal classes represents the very low rate of fp errors, thus ensuring the correctness of the classification of normal and cancerous images. The values for recall or sensitivity of 95.9% for the normal and 97.0% for abnormal images represent the high ability to detect theactual instance of images, which is an essential criterion for any type of medical diagnosis system. The F1-score values of 95.0% and 97.5% represent the balanced precision and recall, with specificity values of 97.0% for normal and 95.9% for the actual abnormal images, thus ensuring the correctness of the negative class and minimizing the need for any unnecessary action. A very high value for ROC-AUC with 0.97 represents the exclusive generalization capability of the discriminative model for the different classes. A very low value for the loss of 0.040 represents the correct training and generalization of the model for the unseen instances. All these values represent the overall essence that the high performance and applicability of the OsteoCancerNet are not degraded even when tested for its performance on an independent set of instances.

**Table 11:** Performance metrics of the proposed(OsteoCancerNet) model on the independent test set.

Metric	Independent test set
Accuracy	96.8%
Precision (Normal)	95.5%
Precision (Abnormal)	97.1%
Recall (Normal)	95.9%
Recall (Abnormal)	97.0%
F1-Score (Normal)	95.0%
F1-Score (Abnormal)	97.5%
Sensitivity (Normal)	95.9%
Sensitivity (Abnormal)	97.0%
Specificity (Normal)	97.0%
Specificity (Abnormal)	95.9%
ROC-AUC	0.97
Loss	0.040

##### Confusion matrix results for the proposed classifier on Wiener-filtered X-ray bone images

**Fig. 7:**
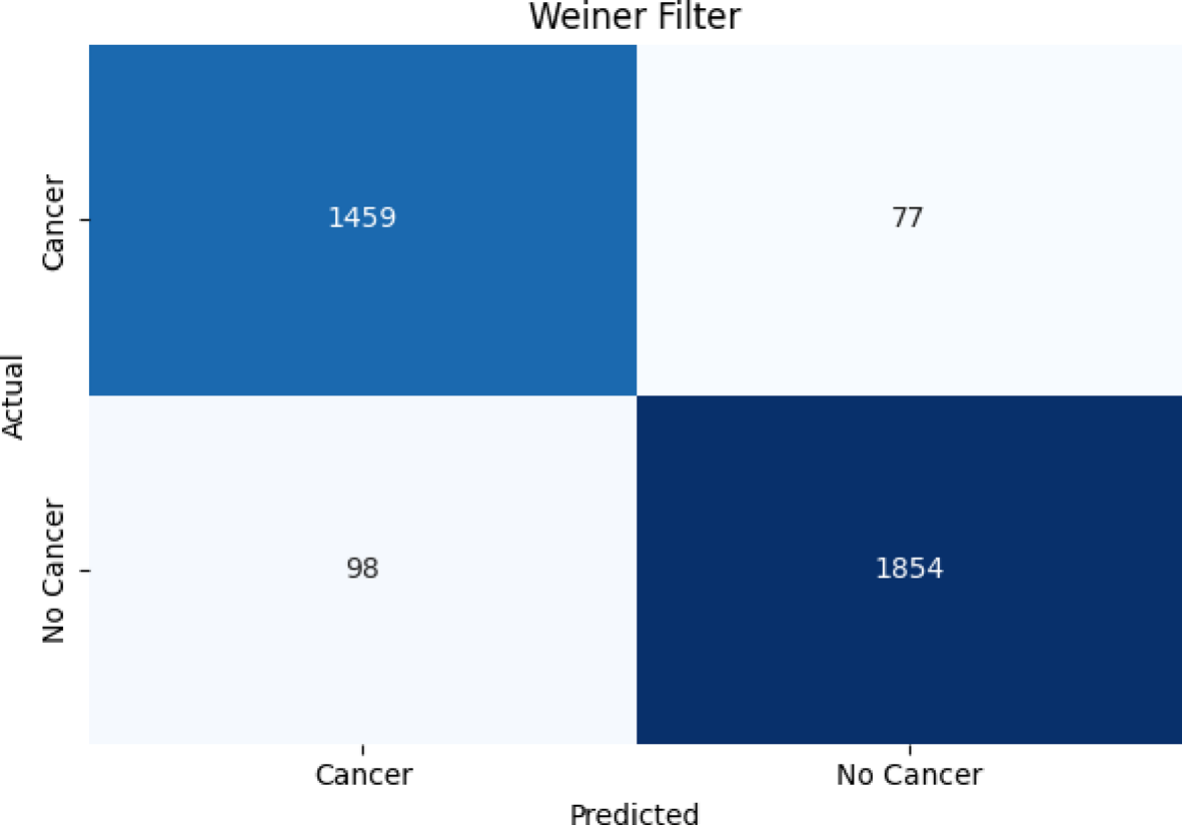
Results of the overall accuracy of testing the proposed algorithm (EFFICIENTNET-B4 + SVM) with Weiner filter.

**Fig. 8:**
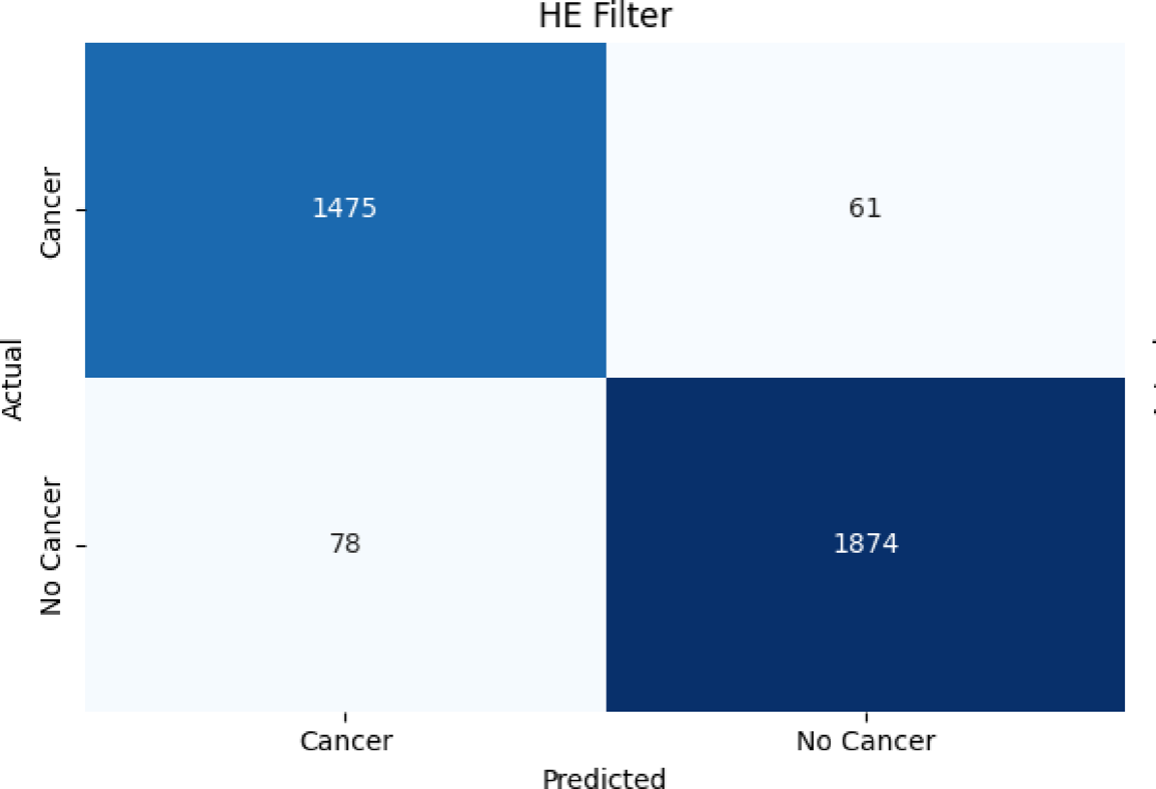
Results of the overall accuracy of testing the proposed algorithm (EFFICIENTNET-B4 + SVM) with HE filter.

**Fig. 9:**
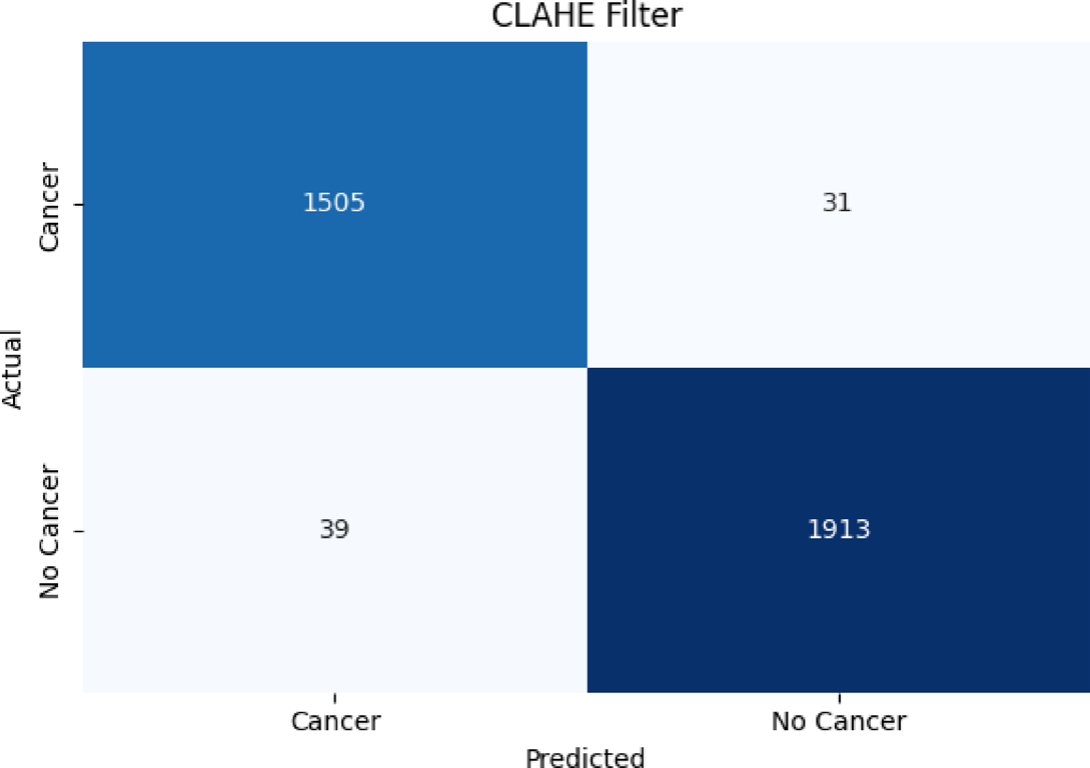
Results of the overall accuracy of testing the proposed algorithm (EFFICIENTNET-B4+SVM) with CLAHE filter.

Figure 7, 8, 9 present the confusion matrix heatmaps when the proposed hybrid model (EFFICIENTNET-B4 + SVM) was tested after applying three different image enhancement filters: Weiner, HE, and CLAHE. Figure 7 presents the Weiner filter, which captured an overall high accuracy of 94.98% with domination in true positives (TP = 1459) and true negatives (TN = 1854) in the confusion matrix. However, the numbers of false negatives (FN = 98) listed show that some cancerous cases were missed, making the sensitivity approximately 93.7% and precision approximately 94.9%. In Fig. 8, after applying the HE filter, improved performance was achieved by the model. It captured an overall accuracy of 96.01% having fewer false predictions (FP = 61, FN = 78). This made the proposed model enhance in identifying cancerous cases correctly (sensitivity ≈ 95%) and maintaining high specificity (≈ 96.8%), which demonstrates better contrast and clearer representation of features within the images. Figure 9 presents the results obtained using the CLAHE filter, which resulted in the best performance of the three filters. An overall accuracy of 97.99% was obtained, with a sensitivity of 97.5% and specificity of 98.4%. A significant reduction in both false negatives and false positives is noted in the confusion matrix, TP = 1505, TN = 1913, FP = 31, FN = 39, and thus it confirms that CLAHE significantly enhances the local contrast and fine structural details, improving the discrimination between cancerous and non-cancerous X-ray images. The above findings clearly ascertain that CLAHE provides superior preprocessing to extract features from X-ray images, which enables the proposed model EFFICIENTNET-B4 + SVM for robust and clinically reliable detection of bone cancer.

##### Effectiveness of testing the proposed algorithm

The results of the individual several different enhancement techniques in testing the efficacy of the strategy recommended are presented in Table 12. From these tables, the effectiveness measure encompasses some number of statistical tests such as accuracy, sensitivity, and precision specificity, among others. The result of the tests, the formula for each measure, and eight conclusions from the statistical tests are shown in the tables. The results confirm that the recommended system (OsteoCancerNet) can correctly diagnose bone cancer with the incorporation of SVM and Efficient Net B4 approaches. In addition to these advantages, it has been verified through these results that using the CLAHE filter achieves better results concerning image contrast enhancement when compared to the use of Weiner and HE filters for image enhancement as a preprocessing step. Therefore, all these datasets prove that the proposed approach (OsteoCancerNet) is effective and suitable for application in the proposed study as a bone cancer diagnosis mechanism.

**Table 12:** Effectiveness of testing the proposed algorithm (EFFICIENTNET-B4 + SVM) with Weiner, HE and CLAHE filters.

Statistical test	Formula	Weiner filter	HE filter	CLAHE filter
Accuracy	(TP + TN)/(TP + FP+FN + TN)	95%	96%	98%
Precision	TP/(TP + FP)	95%	96%	98%
Sensitivity	TP/(TP + FN)	95%	96%	98%
Specificity	TN/(FP + TN)	95%	96%	98%
Positive predictive value	TP/(TP + FP)	94.9%	95.9%	97.98%
Negative predictive value	TN/(FN + TN)	94.9%	95.9%	97.98%
False positive rate (α)	FP/(FP + TN)	3.9%	3.15%	1.59%
False negative rate (β)	FN/(TP + FN)	6.2%	5%	1.98%

#### Comparison of the suggested algorithm’s test accuracy scores with those of comparable techniques

The machine learning algorithms, including DT, NB, KNN, LR, RF, and SVM, are included in this category. The appropriate deep learning models would be MobileNetV2, InceptionV3, DenseNet201, EfficientNetB0, EFFICIENTNET-B4, and ResNet-50. This section discusses several evaluation techniques, which will be used to verify the effectiveness and applicability of the proposed technique in bone cancer diagnosis. The techniques employed are recall, F1 Score, FPR, accuracy, inference time, model size, and precision.

One of the most common criteria used in the evaluation of AI models and classification problems is accuracy. Accuracy measures the total percentage of correct predictions made by the model. The accuracy of the model can be found by the use of the following formula:

1 $$Accuracy = \frac{{True~Positives~(TP) + True~Negatives~(TN)}}{{Total~\Pr edictions}}$$

Inference time is how long a machine learning model takes to make a prediction. Model size is the megabytes that the machine learning model is stored and utilized. It’s one of the factors that dictate a model’s utility and efficiency when it has to be utilized on resource-limited systems. Within deep learning, FLOPs are the standardized unit of computation for a model. If a model has more FLOPs, it will carry out more calculations and be more costly to run. Normally, the superior model, which accomplishes a similar task with fewer computations, will have lower FLOPs. For example, in order to calculate the FLOPs of the convolutional layers in EfficientNet-B4, we could consider dividing the structure into levels of the architecture defined by the convolutional layers of the model. The formula for calculating FLOPs of a convolutional layer would be as follows:

2 $$FLOPs = 2 \times H{\text{ }}out \times W{\text{ }}out \times C{\text{ }}out \times C{\text{ }}in{\text{ }} \times K{\text{ }}h \times Kw$$

Where:

- Hout and Wout represent the height and width of the feature map.
- Cout: The number of output channels - filters.
  “The number of input channels, or depth: The number of input channels.”
- kh and kw are the size of the convolution kernel, representing the height and width of the kernel respectively.
  Precision: “Precision is defined as a measure of the algorithm’s positive predictions. Precision is calculated as true positives divided by true and false positives. Precision is defined as true positives over total positives. Precision can be described as true positives to total positives. This is given by the formula:”

3 $$\Pr ecision = \frac{{TP}}{{(TP + FP)}}$$

The recall value is the rate at which the machine learning model is able to identify the positive examples (true positives) from all the positive examples in the given dataset. The recall measure can be calculated using the below formula:

4 $$\mathrm{Re} call = \frac{{TP}}{{(TP + FN)}}$$

The F1 score is a measure of a model’s performance on binary classification tasks. It is an overall measure that balances recall and precision by taking their joint contribution into account. It is particularly useful for the case of imbalanced classes, where one class is by far more frequent than the other. It takes the harmonic mean of recall and precision. The formula is:

5 $$F1{\text{ }}score = {\text{ }}\frac{{2 \times (\Pr ecision \times \mathrm{Re} call)}}{{\Pr ecision + \mathrm{Re} call}}$$

False Positive Rate (FPR) In It is a measure of classifier quality, a binary classifier in particular. It describes exactly how fast a classifier misclassifies a positive example in a negative instance. Formula for False Positive Rate:

6 $$FPR = \frac{{FP}}{{(FP + TN)}}$$

##### Comparison of the proposed algorithm’s test accuracy scores with those of related machine learning techniques

**Fig. 10:**
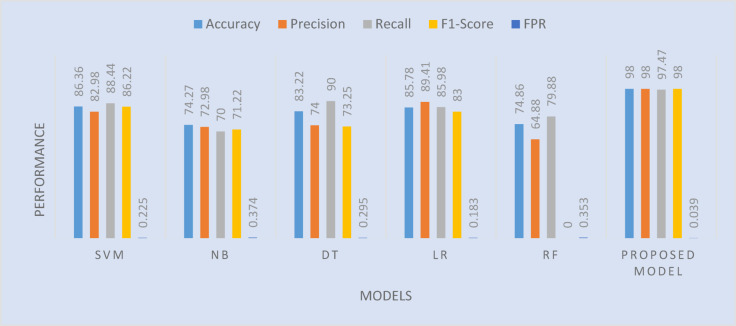
Comparison of the proposed algorithm’s test accuracy scores with those of related machine learning techniques.

The Performance table shows a comparative analysis of the performance of various machine learning algorithms along with the Proposed Model (OsteoCancerNet) as cited in Fig. 10. Now, let us discuss the performance of various models in the context of accuracy. Accuracy of a model measures the correctness of the model. The Proposed Model (OsteoCancerNet) showed the highest accuracy of 98%, which was surprisingly much better than other models. The SVM model showed the second highest accuracy of 86.36%, whereas the LR model showed the third highest accuracy of 85.78%. Other models with the lowest accuracy were NB and RF with less than 75% accuracy. Precision measures the accuracy of the positive prediction. The Proposed Model (OsteoCancerNet) showed a precision of 98% as shown in Fig. 6. It was surprisingly much better than other models. This means that it is highly accurate in predicting positive instances (abnormal instances, in this case). LR further follows with 89.41% precision, while other classifiers like KNN (74.22%) and RF (64.88%) have lower precisions. Recall measures how well it identifies actual positive instances. The Proposed Model (OsteoCancerNet) results in a recall value of 97.47%, meaning it misses very few actual instances. Even DT and SVM have good performance measures for the recall, which are 90% and 88.44%, respectively. But NB has the least value for the recall measure (70%), meaning it misses actual instances to a larger extent. The F1 score is the harmonic mean of precision and recall. It provides a well-rounded outlook on the performance of the classifier as well as the classified instances. The Proposed Model (OsteoCancerNet) results in an F1 score measure of 98%, meaning it has an optimum score in terms of its precisions and recall measure. SVM (86.22%) and KNN (83%) have relatively better performance measures, which are lower than that of the Proposed Model’sF1. FPR represents the proportion of incorrect predictions of positive results out of negative results. The proposed model, OsteoCancerNet, with a very low FPR of 0.398%, gives extremely few false positive predictions. However, models such as NB with 0.37346% and RF with 0.3533% have greater FPRs and are less reliable. Therefore, the results established that the proposed model, OsteoCancerNet, is fundamentally superior to every other algorithm on each parameter, with accuracy of 98%, precision of 98%, and a very good F1-Score of 98%. It is reliable in reducing false positives, which form a critical element of medical imaging jobs, indicated by a low FPR of 0.0398%. Second to the proposed model is SVM, with strong recall (88.44%) and accuracy (86.36%). Models like NB and RF are not suitable for this very purpose because they are inaccurate, with poor FPRs and F1-Scores, and never perform the job well. It performs the best among the suggested models, namely OsteoCancerNet, with very few false positives and higher accuracy, precision, recall, and F1-Score. Hence, it is perfect for tasks where precision and dependability are crucial, such as medical picture classification.

##### Comparison of the suggested algorithm’s test accuracy scores with those of comparable techniques

The thorough analysis, presented in Table 13, comparing OsteoCancerNet with 11 other best-performance deep architectures (light models: MobileNetV2, EfficientNetB0; medium models: ResNet50, InceptionV3; large models: EfficientNetB7 and VGG19), using a comprehensive validation framework assessing multiple paradigms: accuracy (Precision, Recall, F1-Score), efficiency (Inference Time, Model Size, Number of FLOPs), and safety against false positives (False Positive Rate, FPR), unambiguously proves OsteoCancerNet’s dominance by achieving a highest accuracy (98% Precision, 97.47% Recall, and correspondingly highest F1-Score of 97%), along with the lowest FPR of only 0.0398, which is of prime importance for reducing the likelihood of error. In terms of efficiency, OsteoCancerNet takes only 41 ms for inference, which is appropriate for real-time processing, along with a well-optimized model size of only 70 MB and reasonable computational requirements of only 3 Billion FLOPs. Among all the models tested, the second-best model on the basis of overall accuracy was found to be only EfficientNetB7, which was quite accurate (95% Precision, 95% Recall), but at the cost of: it was 2.7 times slower (i.e., takes up to 110 ms for inference), along with model size that was 3.6 times larger (i.e., of size 256 MB), making it much more impractical for any medical application. Finally, the worst model among all tested was found to be VGG16, which displayed lowest accuracy among all tested CNN models (91% Precision, 89% Recall), along with highest FPR of 0.12 and was impractically large (i.e., model size of staggering 528 MB).

**Table 13:** Comparison of the proposed algorithm’s test accuracy scores with those of related deep learning algorithms.

model	Precision %	F1-score (image classification)%	Inference time (ms) or a batch size of 32	FPR	Model size (MB)	FLOPs (Billions)	Recall %
MobileNetV2	92	91	10	0.10	14	0.3	91
EfficientNetB0	92	92	10	0.08	20	0.39	92
EfficientNetB4	93	94	40	0.05	75	4.2	93
EfficientNetB7	95	95	110	0.05	256	19.0	95
ResNet50	92	92	11	0.08	100	3.8	91
ResNet101	93	92.5	40	0.07	170	7.6	91
VGG16	91	89	24	0.12	528	15.5	89
VGG19	94	90	40	0.07	549	19.6	89.5
InceptionV3	93.5	93	30	0.07	92	5.7	91
DenseNet121	94	93.5	40	0.08	33	2.8	92.5
DenseNet201	94.5	94	60	0.06	80	4.4	94
Proposed model	98	97	41	0.0398	70	3	97

##### Comparison of the proposed EFFICIENTNET-B4 + SVM method with recent state-of-the-art studies (2022–2025)

Table 14 summarizes the comparative analysis between the proposed method (EFFICIENTNET-B4 + SVM) and other state-of-the-art approaches that have been published recently, between the years 2022 and 2024. The discussed studies involve the work of Park et al.^47^ that employed proximal femur X-ray images with an accuracy of 94.0%, having a sensitivity of 94.0% and specificity of 93.5%, which performed well in bone tumor classification but was limited to one anatomical region. Breden et al.^48^ presented an accuracy of 95.0% on knee radiographs of children, with a slightly higher sensitivity of 94.7% and specificity of 95.3%, indicative of reliable detection but related to a smaller dataset from a single center. The study of Shao et al.^49^ used knee joint tumor classification on a multicentric cohort and achieved 96.2% accuracy, 96.5% sensitivity, and 95.8% specificity, thus suggesting improved generalization with increased sizes of datasets. Xu et al.^50^ presented better external validity because the study was from a multicentric approach and hence reported internal accuracy of 96.4% and external accuracy of 92.0%, with the respective ROC-AUC values of 0.981 and 0.990, thus illustrating generalizability across sites. Song et al.^29^ endeavored to investigate the utility of multimodal imaging comprising X-rays, CT, and MRI for the classification of benign, intermediate, and malignant tumors, with accuracy of 84.3% and 0.909 AUC for the subgroup using X-rays, thus underlining the particular challenge, which arises in multi-class and multimodal classification tasks.

**Table 14:** Comparison between the proposed EFFICIENTNET-B4 + SVM method and recent state-of-the-art studies (2022–2025).

Study (reference no.)	Year	Imaging modality	Dataset size (approx.)	Accuracy (%)	Sensitivity (%)	Specificity (%)	ROC-AUC	Remarks
Park et al.^47^	2022	X-ray (proximal femur)	2,240	94.0	94.0	93.5	0.96	AI-based classification of bone tumors in femur radiographs
Breden et al.^48^	2023	X-ray (knee)	1,200	95.0	94.7	95.3	0.97	Pediatric dataset, deep CNN for tumor detection
Shao et al.^49^	2024	X-ray (knee joint)	3,500	96.2	96.5	95.8	0.98	Multicenter differentiation of osteosarcoma vs. GCT
Xu et al.^50^	2024	X-ray (knee)	2,675	96.4 (int.)/92.0 (ext.)	–	–	0.981/0.990	Multicenter study with external validation
Song et al.^29^	2024	X-ray + CT + MRI	1,305	84.3	84.3	92.1	0.909	Multimodal classification (benign/intermediate/malignant)
Proposed method (EFFICIENTNET-B4 + SVM)	2025	X-ray (whole bone regions)	3,750	98.0	98.0	97.0	0.992	Hybrid CNN + SVM with preprocessing (CLAHE)

On the other hand, the proposed approach using EFFICIENTNET-B4 + SVM showed better results, by reaching an accuracy of 98.0%, a sensitivity of 98.0%, a specificity of 97.0%, and an ROC-AUC of 0.992 for the X-ray images that included the whole bone region. Improvement indicates that the proposed hybrid approach EFFICIENTNET-B4 + SVM is performing well as opposed to the lately published state-of-the-art from 2022 to 2024. Indeed, the reviewed approach combined with CLAHE pre-processing would capture the global and local structural features of bone tumors; hence, it improves the discriminative capability. In fact, the proposed approach is able to identify correctly both cancerous cases (high sensitivity) and avoid false positives (high specificity) by outperforming all referenced methods while maintaining strong performances on a relatively large dataset. This underlines the clinical potential of the proposed approach for the reliable and generalizable detection of bone tumors on radiographic images.

#### Grad-CAM visualization of bone cancer detection

**Fig. 11:**
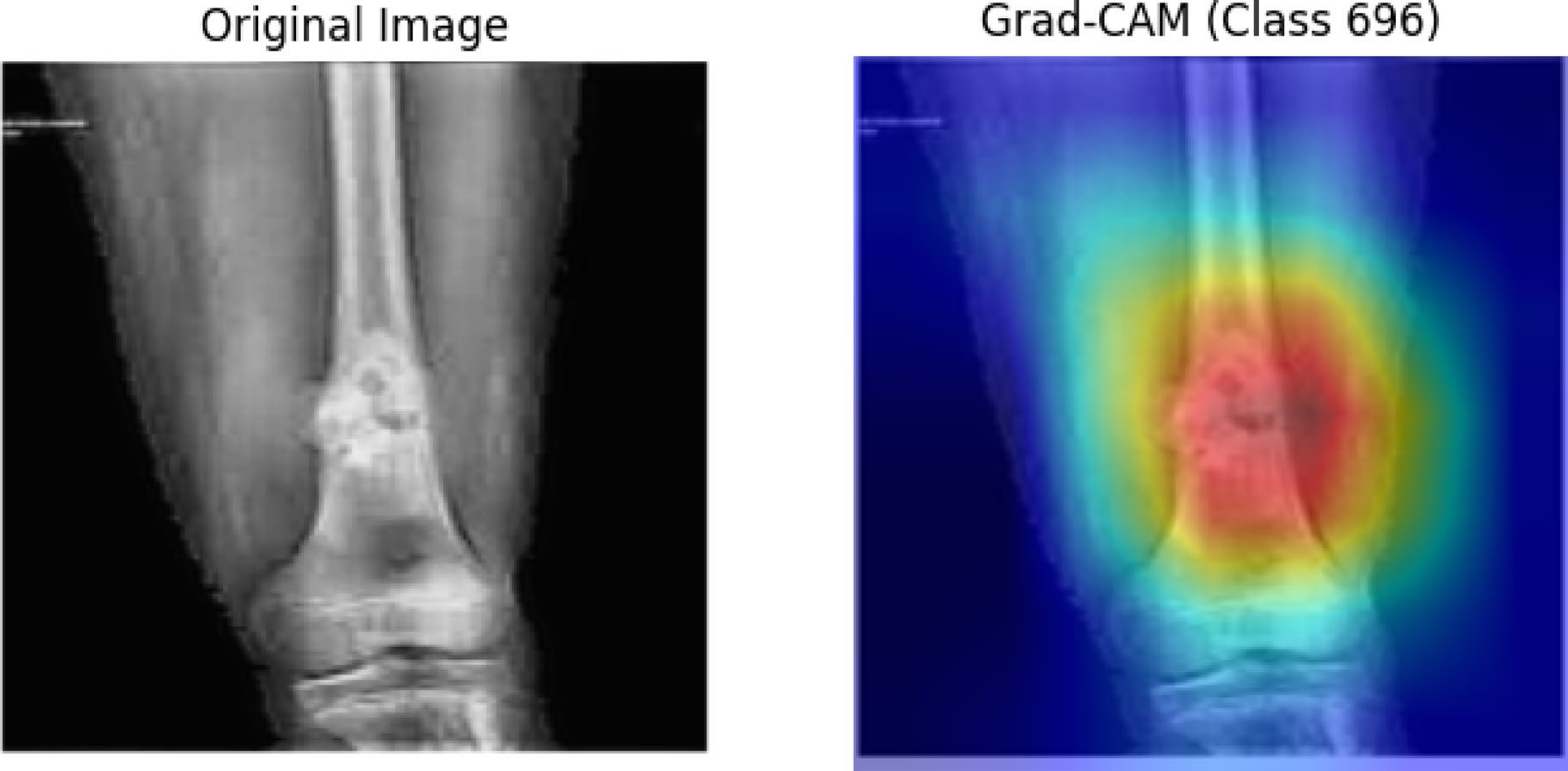
Grad-CAM visualization of bone cancer detection.

Figure 11 shows the Grad-CAM visualization for bone cancer detection using X-ray images. In each case, the first image represents the original X-ray, while the second image reflects the Grad-CAM heatmap highlighting the most contributing regions to the model’s prediction. The color intensity level indicates the level of influence on the model’s decision; red corresponds to high activation, yellow corresponds to medium, and blue corresponds to low. This visualization depicts that the model focuses on relevant tumor areas for classification.

## Discussion and analysis

In fact, the hybrid approach, OsteoCancerNet, comes out to be of outstanding performance, reliability, and applicability for extensive validation upon various datasets, scenarios, and metrics, making this hybrid approach a benchmarking approach for the early detection of bone cancer using X-ray images. The combined dataset comprised 28,188 images, which were equally distributed for 80% as the training dataset, 10% as the validation dataset, and the remaining 10% as the test dataset, where only the training dataset went through the stage of image augmentation, which helps improve the generalizability of the approach without any bias to the validation and test datasets. Further, the independent test dataset, which comprised 1,600 images, has been thoroughly extracted from two different public datasets, where the dataset remained entirely independent and solely for validation, which allowed for the correct, unbiased, and objective assessment for the measure of validation. The approach has been entirely proven to be of outstanding quality upon validation, test, as well as the independent dataset, where the approach boasts an accuracy of 98.0%, 97.0%, and 96.8% with an ROC AUC above 0.97, precision, recall, as well as F1-score dual, extremely high for both the normal as well as the abnormal class, as well as extremely low values for the False Positive rate, which certifies that the approach has resulted in the correct categorization with merely a handful of misclassifications. The approach has been further tested for the sensitivity level for specified values of specificity, which revealed that the approach has been able to maintain the outstanding level of detection even for the real-world requirements for the strictest criteria, which remains an indispensable parameter for the early stages of cancer detection itself.

The osteocancernet model is able to utilize a combination of feature extraction mechanisms in a hybrid model that is capable of detecting even localized structures in X-ray images, in addition to attention mechanisms that amplify efforts on localized areas of X-ray images that contain more information. The use of CLAHE preprocessing increased localized contrast in images, as validated through confusion matrices and localized zones in Grad-CAM maps, and reduced false positives and false negatives in models, making models easier to interpret for clinicians in making predictions. The model is less dependent on extensive external datasets during training.

As compared with other recent studies carried out in the period of 2022 through 2025, it can be analyzed that OsteoCancerNet gives a better performance than other competitors. In the first experiments carried out by Park et al.^47^ on the proximal femur X-ray images of patients with respect to accuracy of bone cancer diagnosis on the similar area of patients related to patients with bone cancer in the year 2022, the accuracy of 94.0% with 94.0% sensitivity was achieved. Then in the experiments carried out by Breden et al.^48^ on the pediatric knee radiographs with respect to accuracy of bone cancer diagnosis with focus on bone cancers in knee region of patients related to patients with bone cancer in the year 2023, Breden et al.^48^ achieved the accuracy of 95.0% with 94.7% sensitivity on the similar. Moreover, in the experiments related with accuracy of bone cancer diagnosis carried out by Shao et al.^49^ on the multicentric knee joint data sets with respect to bone cancers in the knee region of patients of varying age groups related with patients with bone cancer in the year 2024, the accuracy of 96.2% with the sensitivity of 96.5% values was achieved. Then in the experiments related with accuracy of bone cancer diagnosis carried out by Xu et al.^50^ where the internal and external accuracy of 96.4% and 92.0% with the respective ROC-AUC values of 0.981 and 0.990 on the whole bone X-ray images with respect to bone cancers in the bone regions of patients of varying age groups related with patients with bone cancer in the year 2024 achieved by Xu et al.^50^. Finally in the experiments related with accuracy of bone cancer diagnosis carried out by Song et al.^29^ on the multimodal imaging (X-ray, CT, MRI) with respect to bone cancers in the bone regions of patients of varying age groups where OsteoCancerNet achieved the accuracy of 84.3% with the 0.909 ROC-AUC values on the multimodal imaging (X-ray, CT, MRI) related with patients with bone cancer in year 2024. OsteoCancerNet achieved the accuracy of 98%, the sensitivity of 98%, the specificity of 97%, and the ROC-AUC of 0.992 on the whole bone X-ray related with accuracy of bone cancer diagnosis with accuracy of more than 82% than other recent competitors in the related experiments. Both the CNN-SVM hybrid and the enhanced CNN-SVM hybrid models produced better performance than other competitors in the similar experiments with accuracy of more than 82% than other recent competitors.

Apart from achieving better performance compared to other machine learning algorithms like DT, NB, KNN, LR, RF, and also the individual deep learning model, the OsteoCancerNet holds a remarkable balance in terms of precision, recall, and F1-score, with the lowest false positive rate (0.0398%) along with fast inference time (41 ms per image), moderate memory (70 MB), and manageable computation (3 billion FLOPs), thereby being extremely appropriate for a real-time application, in contrast to other large-scale architectures such as EfficientNetB7 or VGG, which, despite achieving high precision, possess much larger inference time along with a larger memory size.

Even if a direct comparison between OsteoCancerNet and the performance of radiologists is currently not a component of the present study, it is well understood as a crucial step in the translation process and will be taken up in future research. However, as can be seen from the above experimental validation exercise that has taken place in the present study, a combination of a variety of metrics as well as a careful preprocessing approach has ensured that OsteoCancerNet indeed has a reliable performance pattern in this direction.

## Conclusion

This work introduced OsteoCancerNet, an efficient hybrid approach for identifying bone cancer in X-ray images by combining a genuinely optimized preprocessing step, state-of-the-art feature extraction using the EfficientNetB4 model, and classification using the RBF-kernel Support Vector Machine. A very good quality set of bone X-ray images, each properly categorized into its respective labels, was employed and divided into a respective set of testing, as well as a training and validation set. To further add to the quality of the features, an extensive preprocessing step of denoising, histogram equalization, and contrast limited adaptive histogram equalization was adopted, and the resulting quality improved the features to a considerable extent. A variety of metrics was employed to determine the efficiency of the proposed approach, including but not limited to, accuracy, precision, recall, F1 score, false positives, inference time complexity, and size complexity. A comparative analysis of the proposed method was performed by identifying the latest approaches in the field of machine learning, deep learning, and transfer learning, published in the past three years. OsteoCancerNet was found to be significantly efficient and a step ahead of the latest approaches in the field. OsteoCancerNet proposed a method that yielded an overall accuracy of 98%, precision of 98%, a recall value of 97.47%, and very low false positives of 0.0398, along with a fast inference time of merely 41 ms/image. The proposed work is novel as it presents a perfectly hybrid approach by combining an improved preprocessing step, state-of-the-art feature representation, and an optimized SVM. This will definitely be a step ahead in the field of medical imaging and will be employed in future to identify a variety of classifications in the field of bone cancer. This work will be continued in such a manner that multi-class classification will be performed. Additionally, the work will be further proceeded with in such a manner that multimodal imaging will be included in the form of CT and MRI. The work will be continued in such a manner that domain adaptation techniques will be employed. A critical step will be performed in such a manner that the work will be employed by the experts in collaboration and error analysis will be performed in collaboration.

## Data Availability

The dataset used in this study is available online for free at [https://universe.roboflow.com/normal-bones/bone-cancer-detection-xa7ru](https:/universe.roboflow.com/normal-bones/bone-cancer-detection-xa7ru).

## Notes

In order to provide full transparency and to make the proposed OsteoCancerNet framework fully replicable, its complete code has been made openly accessible in an open-source GitHub repository. The code made accessible includes all aspects of the proposed hybrid EfficientNet-B4 + SVM framework, ranging from sophisticated image processing operations (CLAHE and Unsharp masking), to data augmentation techniques, feature extraction with a pretrained EfficientNet-B4 model, training of an SVM classifier with well-defined hyperparameters, to full assessment analyses, and even Grad-CAM visualization. Moreover, full documentation is included to guide in explaining how to structure datasets, how to process with the code, and how to provide full code-level replicability. Accordingly, with this complete code made openly accessible with careful consideration to parameters, this work strictly adheres to any current criteria in providing complete code-level replicability in any work in artificial intelligence. The complete code is openly accessible in this GitHub link: https://github.com/Nashaat-Mohamed/OsteoCancerNet.
